# (Benzyl­diphenyl­phosphine-3κ*P*)[μ-bis(diphenyl­arsino)methane-1:2κ^2^ *As*:*As*′]nona­carbonyl-1κ^3^ *C*,2κ^3^ *C*,3κ^3^ *C*-*triangulo*-triruthenium(0)

**DOI:** 10.1107/S1600536809053045

**Published:** 2009-12-16

**Authors:** Omar bin Shawkataly, Imthyaz Ahmed Khan, Chin Sing Yeap, Hoong-Kun Fun

**Affiliations:** a Chemical Sciences Programme, School of Distance Education, Universiti Sains Malaysia, 11800 USM, Penang, Malaysia; b X-ray Crystallography Unit, School of Physics, Universiti Sains Malaysia, 11800 USM, Penang, Malaysia

## Abstract

The asymmetric unit of the title *triangulo*-triruthenium compound, [Ru_3_(C_25_H_22_As_2_)(C_19_H_17_P)(CO)_9_], consists of two crystallographically independent mol­ecules of the *triangulo*-triruthenium complex, *A* and *B*. The bis­(diphenyl­arsino)methane ligand bridges an Ru—Ru bond and the monodentate phosphine ligand bonds to the third Ru atom. Both the phosphine and arsine ligands are equatorial with respect to the Ru_3_ triangle. In addition, each Ru atom carries one equatorial and two axial terminal carbonyl ligands. With regard to the three phosphine-substituted rings, the benzyl ring makes dihedral angles of 41.0 (3) and 43.9 (3)° with the other two benzene rings in mol­ecule *A*; these angles are 49.8 (3) and 56.8 (3)° in mol­ecule *B*. The dihedral angles between the two benzene rings are 76.1 (3) and 88.2 (3)° for the two diphenyl­arsino groups in mol­ecule *A* and 71.3 (3) and 78.1 (3)° in mol­ecule *B*. In the crystal packing, mol­ecules are linked into chains *via* inter­molecular C—H⋯O hydrogen bonds. Weak inter­molecular C—H⋯π inter­actions further stabilize the crystal structure.

## Related literature

For general background to *triangulo*-triruthenium derivatives, see: Bruce *et al.* (1985 ▶, 1988**a* ▶,b* ▶). For related structures, see: Shawkataly *et al.* (1998 ▶, 2004 ▶, 2009 ▶). For the synthesis of μ-bis­(diphenyl­arsino)methane­deca­carbonyl­triruthenium(0), see: Bruce *et al.* (1983 ▶). For stability of the temperature controller used for the data collection, see: Cosier & Glazer (1986 ▶).
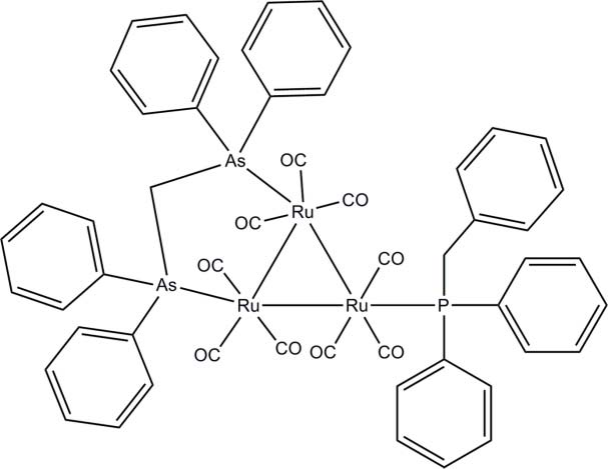


## Experimental

### 

#### Crystal data

[Ru_3_(C_25_H_22_As_2_)(C_19_H_17_P)(CO)_9_]
*M* *_r_* = 1303.86
Monoclinic, 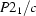

*a* = 13.4824 (2) Å
*b* = 35.5978 (6) Å
*c* = 23.3473 (4) Å
β = 117.597 (1)°
*V* = 9930.5 (3) Å^3^
*Z* = 8
Mo *K*α radiation
μ = 2.31 mm^−1^
*T* = 100 K
0.48 × 0.16 × 0.03 mm

#### Data collection

Bruker SMART APEXII CCD area-detector diffractometer
Absorption correction: multi-scan (*SADABS*; Bruker, 2005 ▶) *T*_min_ = 0.402, *T*_max_ = 0.945
131198 measured reflections
29004 independent reflections
18757 reflections with *I* > 2σ(*I*)
*R*_int_ = 0.071

#### Refinement

*R*[*F* ^2^ > 2σ(*F* ^2^)] = 0.062
*wR*(*F* ^2^) = 0.112
*S* = 1.05
29004 reflections
1226 parameters
H-atom parameters constrained
Δρ_max_ = 2.08 e Å^−3^
Δρ_min_ = −1.19 e Å^−3^

### 

Data collection: *APEX2* (Bruker, 2005 ▶); cell refinement: *SAINT* (Bruker, 2005 ▶); data reduction: *SAINT*; program(s) used to solve structure: *SHELXTL* (Sheldrick, 2008 ▶); program(s) used to refine structure: *SHELXTL*; molecular graphics: *SHELXTL*; software used to prepare material for publication: *SHELXTL* and *PLATON* (Spek, 2009 ▶).

## Supplementary Material

Crystal structure: contains datablocks global, I. DOI: 10.1107/S1600536809053045/sj2709sup1.cif

Structure factors: contains datablocks I. DOI: 10.1107/S1600536809053045/sj2709Isup2.hkl

Additional supplementary materials:  crystallographic information; 3D view; checkCIF report

## Figures and Tables

**Table 1:** Hydrogen-bond geometry (Å, °)

*D*—H⋯*A*	*D*—H	H⋯*A*	*D*⋯*A*	*D*—H⋯*A*
C16*A*—H16*A*⋯O6*B*	0.93	2.59	3.189 (6)	123
C30*A*—H30*A*⋯O7*A*^i^	0.93	2.58	3.261 (6)	130
C42*B*—H42*B*⋯O9*B*^ii^	0.93	2.58	3.241 (7)	129
C28*A*—H28*A*⋯*Cg*1^iii^	0.93	2.72	3.570 (7)	152
C28*B*—H28*B*⋯*Cg*2^iv^	0.93	2.87	3.648 (7)	142
C40*A*—H40*A*⋯*Cg*3	0.93	2.77	3.474 (7)	133
C44*B*—H44*B*⋯*Cg*4	0.93	2.94	3.615 (7)	130

Symmetry codes: (i) 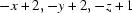; (ii) 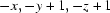; (iii) 
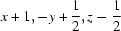
; (iv) 
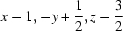
. *Cg*1, *Cg*2, *Cg*3 and *Cg*4 are the centroids of the C1*B*–C6*B*, C1*A*–C6*A*, C26*A*–C31*A* and C26*B*–C31*B* benzene rings, respectively.

**Fig. 1.**
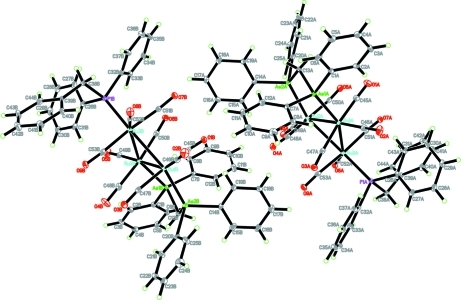
The molecular structure of the title compound with 30% probability ellipsoids for non-H atoms.

**Fig. 2.**
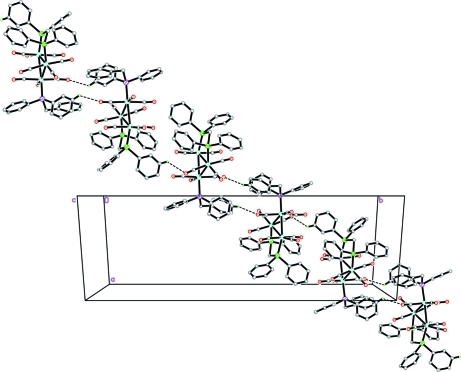
The crystal packing of the title compound, viewed down the c axis, showing the molecules linked into a chain. Hydrogen atoms that not involved in the hydrogen-bonding (dashed lines) have been omitted for clarity.

## supplementary crystallographic information

### Comment

*Triangulo*-triruthenium clusters are known for their interesting structural variations and related catalytic activity. A large number of substituted derivatives, Ru_3_(CO)_12-n_*L*_n_ (*L* = group 15 ligand) have been reported (Bruce, Liddell *et al.*, 1988*a, b*; Bruce *et al.*, 1985). As part of our study on the substitution of transition metal-carbonyl clusters with mixed-ligand complexes, we have published several structures of *triangulo*-triruthenium-carbonyl clusters containing mixed P/As and P/Sb ligands (Shawkataly *et al.*, 1998, 2004, 2009). Herein we report the synthesis and structure of Ru_3_(C_19_H_17_P)(C_25_H_22_As_2_)(CO)_9_.

The asymmetric unit consists of two crystallographically independent molecules of the *triangulo*-triruthenium complex, *A* and *B* (Fig. 1). The bond lengths and angles of title compound are comparable to those found in its related structure (Shawkataly *et al.*, 2009). The bis(diphenylarsino)methane ligand bridges the Ru1—Ru2 bond and the monodentate phosphine ligand bonds to the Ru3 atom. Both the phosphine and arsine ligands are equatorial with respect to the Ru_3_ triangle. Additionally, each Ru atom carries one equatorial and two axial terminal carbonyl ligands. For the three phosphine-substituted rings, the benzyl ring makes dihedral angles of 41.0 (3) and 43.9 (3)° (C26–C31/C39–C44 and C32–C37/C39–C44) with the other two benzene rings in molecule *A* whereas these angles are 49.8 (3) and 56.8 (3)° in molecule *B*. The dihedral angles between the two benzene rings (C1–C6/C7–C12 and C14–C19/C20–C25) are 76.1 (3) and 88.2 (3)° for the two diphenylarsino groups in molecule *A* and 71.3 (3) and 78.1 (3)° in molecule *B*.

In the crystal packing, the molecules are linked into chains *via* intermolecular C16A—H16A···O6B, C30A—H30A···O7A and C42B—H42B···O9B hydrogen bonds (Fig. 2). Weak intermolecular C—H···π interactions further stabilize the crystal structure (Table 1).

### Experimental

All manipulations were performed under a dry oxygen-free dinitrogen atmosphere using standard Schlenk techniques, all solvents were dried over sodium and distilled from sodium benzophenone ketyl under nitrogen. Benzyldiphenylphosphine (Strem Chemicals) was used as recieved and µ-bis(diphenylarsino)methane-decacarbonyl-triruthenium(0) (Bruce *et al.*, 1983) was prepared by a reported procedure. The title compound was obtained by refluxing equimolar quantities of Ru_3_(CO)_10_(µ-Ph_2_AsCH_2_AsPh_2_) (105.5 mg, 0.1 mmol) and benzyldiphenylphosphine (32.02 mg, 0.1 mmol) in hexane under a nitrogen atmosphere. Crystals suitable for X-ray diffraction were grown by slow solvent / solvent diffusion of CH_3_OH into CH_2_Cl_2_.

### Refinement

All hydrogen atoms were positioned geometrically and refined using a riding model with C—H = 0.93–0.97 Å and *U*_iso_(H) = 1.2 *U*_eq_(C). There are several relatively large peaks in the the difference map. However, a suitable alternative model based on the solvents used in the synthesis or the crystallization of the title complex could not be found. The maximum and minimum residual electron density peaks of 2.08 and -1.19 eÅ^-3^, respectively, were located 1.19 Å and 0.90 Å from the C7A and RU3A atoms, respectively.

### Crystal data

**Table:**

[Ru_3_(C_25_H_22_As_2_)(C_19_H_17_P)(CO)_9_]	*F*(000) = 5136
*M**_r_* = 1303.86	*D*_x_ = 1.744 Mg m^−^^3^
Monoclinic, *P*2_1_/*c*	Mo *K*α radiation, λ = 0.71073 Å
Hall symbol: -P 2ybc	Cell parameters from 9933 reflections
*a* = 13.4824 (2) Å	θ = 2.3–29.2°
*b* = 35.5978 (6) Å	µ = 2.31 mm^−^^1^
*c* = 23.3473 (4) Å	*T* = 100 K
β = 117.597 (1)°	Plate, red
*V* = 9930.5 (3) Å^3^	0.48 × 0.16 × 0.03 mm
*Z* = 8	

### Data collection

**Table:**

Bruker SMART APEXII CCD area-detector diffractometer	29004 independent reflections
Radiation source: fine-focus sealed tube	18757 reflections with *I* > 2σ(*I*)
graphite	*R*_int_ = 0.071
φ and ω scans	θ_max_ = 30.0°, θ_min_ = 1.1°
Absorption correction: multi-scan (*SADABS*; Bruker, 2005)	*h* = −18→18
*T*_min_ = 0.402, *T*_max_ = 0.945	*k* = −50→44
131198 measured reflections	*l* = −32→22

### Refinement

**Table:**

Refinement on *F*^2^	Primary atom site location: structure-invariant direct methods
Least-squares matrix: full	Secondary atom site location: difference Fourier map
*R*[*F*^2^ > 2σ(*F*^2^)] = 0.062	Hydrogen site location: inferred from neighbouring sites
*wR*(*F*^2^) = 0.112	H-atom parameters constrained
*S* = 1.05	*w* = 1/[σ^2^(*F*_o_^2^) + (0.0284*P*)^2^ + 35.7105*P*] where *P* = (*F*_o_^2^ + 2*F*_c_^2^)/3
29004 reflections	(Δ/σ)_max_ = 0.002
1226 parameters	Δρ_max_ = 2.08 e Å^−^^3^
0 restraints	Δρ_min_ = −1.19 e Å^−^^3^

### Special details

**Table:**

Experimental. The crystal was placed in the cold stream of an Oxford Cyrosystems Cobra open-flow nitrogen cryostat (Cosier & Glazer, 1986) operating at 100.0 (1) K.
Geometry. All e.s.d.'s (except the e.s.d. in the dihedral angle between two l.s. planes) are estimated using the full covariance matrix. The cell e.s.d.'s are taken into account individually in the estimation of e.s.d.'s in distances, angles and torsion angles; correlations between e.s.d.'s in cell parameters are only used when they are defined by crystal symmetry. An approximate (isotropic) treatment of cell e.s.d.'s is used for estimating e.s.d.'s involving l.s. planes.
Refinement. Refinement of *F*^2^ against ALL reflections. The weighted *R*-factor *wR* and goodness of fit *S* are based on *F*^2^, conventional *R*-factors *R* are based on *F*, with *F* set to zero for negative *F*^2^. The threshold expression of *F*^2^ > σ(*F*^2^) is used only for calculating *R*-factors(gt) *etc*. and is not relevant to the choice of reflections for refinement. *R*-factors based on *F*^2^ are statistically about twice as large as those based on *F*, and *R*- factors based on ALL data will be even larger.

### Fractional atomic coordinates and isotropic or equivalent isotropic displacement parameters (Å^2^)

**Table:**

	*x*	*y*	*z*	*U*_iso_*/*U*_eq_	
Ru1A	0.76373 (3)	0.886190 (11)	0.500136 (19)	0.01238 (9)	
Ru2A	0.65234 (3)	0.851053 (11)	0.375632 (19)	0.01265 (9)	
Ru3A	0.88866 (3)	0.859273 (11)	0.436070 (19)	0.01141 (8)	
As1A	0.59031 (4)	0.886381 (14)	0.50761 (2)	0.01318 (11)	
As2A	0.46232 (4)	0.864590 (14)	0.35505 (2)	0.01289 (11)	
P1A	1.07682 (10)	0.86564 (4)	0.51268 (6)	0.0133 (3)	
O1A	0.7068 (3)	0.96100 (10)	0.4261 (2)	0.0276 (9)	
O2A	0.9059 (3)	0.92692 (11)	0.62601 (19)	0.0306 (10)	
O3A	0.8498 (3)	0.81485 (10)	0.58225 (18)	0.0223 (8)	
O4A	0.6528 (3)	0.78247 (10)	0.45562 (18)	0.0237 (9)	
O5A	0.6323 (3)	0.80072 (11)	0.26561 (19)	0.0305 (10)	
O6A	0.6353 (3)	0.92231 (11)	0.29746 (19)	0.0277 (9)	
O7A	0.8800 (3)	0.93986 (10)	0.38671 (17)	0.0179 (8)	
O8A	0.9127 (3)	0.83289 (10)	0.3200 (2)	0.0262 (9)	
O9A	0.8950 (3)	0.77640 (10)	0.47621 (18)	0.0223 (8)	
C1A	0.5646 (4)	0.92845 (14)	0.5528 (2)	0.0165 (11)	
C2A	0.6195 (4)	0.96206 (14)	0.5569 (2)	0.0172 (11)	
H1	0.6700	0.9635	0.5402	0.021*	
C3A	0.6007 (5)	0.99346 (14)	0.5852 (3)	0.0205 (12)	
H2	0.6388	1.0157	0.5881	0.025*	
C4A	0.5245 (4)	0.99142 (16)	0.6092 (3)	0.0223 (12)	
H3	0.5111	1.0124	0.6283	0.027*	
C5A	0.4685 (4)	0.95852 (16)	0.6050 (3)	0.0231 (12)	
H4	0.4170	0.9575	0.6210	0.028*	
C6A	0.4878 (4)	0.92695 (16)	0.5773 (3)	0.0229 (12)	
H5	0.4497	0.9047	0.5749	0.027*	
C7A	0.5601 (4)	0.84357 (14)	0.5489 (3)	0.0172 (11)	
C8A	0.6318 (4)	0.83769 (14)	0.6143 (3)	0.0204 (12)	
H6	0.6905	0.8543	0.6369	0.025*	
C9A	0.6155 (5)	0.80729 (16)	0.6456 (3)	0.0282 (14)	
H7	0.6624	0.8036	0.6895	0.034*	
C10A	0.5294 (5)	0.78217 (16)	0.6117 (3)	0.0282 (14)	
H10A	0.5186	0.7617	0.6329	0.034*	
C11A	0.4599 (5)	0.78746 (15)	0.5468 (3)	0.0264 (13)	
H11A	0.4032	0.7702	0.5243	0.032*	
C12A	0.4733 (4)	0.81818 (14)	0.5147 (3)	0.0209 (12)	
H12A	0.4252	0.8219	0.4711	0.025*	
C13A	0.4541 (4)	0.89210 (13)	0.4250 (2)	0.0149 (10)	
H13A	0.3904	0.8831	0.4299	0.018*	
H13B	0.4422	0.9185	0.4139	0.018*	
C14A	0.3564 (4)	0.82396 (14)	0.3375 (2)	0.0166 (11)	
C15A	0.3831 (4)	0.78765 (13)	0.3276 (2)	0.0152 (11)	
H15A	0.4531	0.7826	0.3307	0.018*	
C16A	0.3058 (4)	0.75911 (14)	0.3130 (2)	0.0188 (11)	
H16A	0.3244	0.7347	0.3074	0.023*	
C17A	0.2004 (4)	0.76683 (15)	0.3067 (3)	0.0221 (12)	
H17A	0.1482	0.7476	0.2967	0.026*	
C18A	0.1729 (5)	0.80301 (15)	0.3154 (3)	0.0277 (14)	
H18A	0.1024	0.8082	0.3113	0.033*	
C19A	0.2504 (4)	0.83151 (15)	0.3301 (3)	0.0259 (13)	
H19A	0.2313	0.8559	0.3351	0.031*	
C20A	0.3729 (4)	0.89564 (14)	0.2808 (2)	0.0171 (11)	
C21A	0.3098 (4)	0.92572 (15)	0.2820 (3)	0.0224 (12)	
H21A	0.3100	0.9327	0.3205	0.027*	
C22A	0.2450 (5)	0.94595 (16)	0.2250 (3)	0.0303 (14)	
H22A	0.2036	0.9666	0.2262	0.036*	
C23A	0.2424 (5)	0.93566 (17)	0.1681 (3)	0.0290 (14)	
H23A	0.2003	0.9494	0.1307	0.035*	
C24A	0.3029 (5)	0.90466 (16)	0.1664 (3)	0.0274 (13)	
H24A	0.2993	0.8971	0.1274	0.033*	
C25A	0.3686 (4)	0.88493 (16)	0.2222 (3)	0.0243 (12)	
H25A	0.4102	0.8644	0.2207	0.029*	
C26A	1.1224 (4)	0.90445 (13)	0.5703 (2)	0.0140 (10)	
C27A	1.2271 (4)	0.90382 (15)	0.6253 (3)	0.0196 (11)	
H27A	1.2702	0.8821	0.6355	0.024*	
C28A	1.2671 (4)	0.93504 (15)	0.6644 (3)	0.0231 (12)	
H28A	1.3364	0.9340	0.7010	0.028*	
C29A	1.2053 (4)	0.96770 (15)	0.6498 (3)	0.0214 (12)	
H29A	1.2334	0.9888	0.6760	0.026*	
C30A	1.1011 (5)	0.96911 (14)	0.5959 (3)	0.0214 (12)	
H30A	1.0588	0.9910	0.5859	0.026*	
C31A	1.0604 (4)	0.93770 (13)	0.5570 (2)	0.0165 (11)	
H31A	0.9901	0.9387	0.5212	0.020*	
C32A	1.1319 (4)	0.82436 (13)	0.5641 (3)	0.0162 (11)	
C33A	1.1788 (4)	0.79435 (14)	0.5468 (3)	0.0232 (12)	
H33A	1.1893	0.7962	0.5103	0.028*	
C34A	1.2098 (5)	0.76191 (16)	0.5831 (3)	0.0294 (14)	
H34A	1.2413	0.7422	0.5710	0.035*	
C35A	1.1942 (5)	0.75856 (15)	0.6374 (3)	0.0285 (14)	
H35A	1.2152	0.7366	0.6618	0.034*	
C36A	1.1470 (4)	0.78813 (15)	0.6554 (3)	0.0230 (12)	
H36A	1.1362	0.7861	0.6918	0.028*	
C37A	1.1163 (4)	0.82044 (14)	0.6186 (3)	0.0204 (12)	
H37A	1.0844	0.8401	0.6306	0.024*	
C38A	1.1753 (4)	0.87114 (14)	0.4783 (3)	0.0174 (11)	
H38A	1.2513	0.8711	0.5133	0.021*	
H38B	1.1676	0.8497	0.4508	0.021*	
C39A	1.1569 (4)	0.90676 (14)	0.4392 (3)	0.0174 (11)	
C40A	1.1878 (4)	0.94205 (15)	0.4688 (3)	0.0215 (12)	
H40A	1.2272	0.9438	0.5136	0.026*	
C41A	1.1596 (5)	0.97433 (15)	0.4316 (3)	0.0264 (13)	
H41A	1.1774	0.9977	0.4518	0.032*	
C42A	1.1057 (5)	0.97248 (16)	0.3650 (3)	0.0270 (13)	
H42A	1.0862	0.9944	0.3405	0.032*	
C43A	1.0811 (5)	0.93808 (16)	0.3351 (3)	0.0266 (13)	
H43A	1.0487	0.9367	0.2902	0.032*	
C44A	1.1044 (4)	0.90529 (15)	0.3716 (3)	0.0198 (12)	
H44A	1.0848	0.8821	0.3508	0.024*	
C45A	0.7269 (4)	0.93195 (15)	0.4502 (3)	0.0190 (12)	
C46A	0.8544 (4)	0.91159 (15)	0.5775 (3)	0.0185 (11)	
C47A	0.8161 (4)	0.83991 (15)	0.5474 (3)	0.0170 (11)	
C48A	0.6587 (4)	0.80877 (15)	0.4286 (3)	0.0193 (12)	
C49A	0.6364 (4)	0.82009 (15)	0.3053 (3)	0.0197 (12)	
C50A	0.6473 (4)	0.89623 (15)	0.3288 (3)	0.0198 (12)	
C51A	0.8788 (4)	0.91046 (14)	0.4065 (2)	0.0137 (10)	
C52A	0.9062 (4)	0.84217 (13)	0.3651 (3)	0.0161 (11)	
C53A	0.8882 (4)	0.80748 (15)	0.4629 (2)	0.0161 (11)	
Ru1B	0.42063 (3)	0.640248 (11)	0.520022 (19)	0.01315 (9)	
Ru2B	0.30365 (3)	0.605718 (11)	0.395962 (19)	0.01302 (9)	
Ru3B	0.18372 (3)	0.637768 (11)	0.460014 (19)	0.01191 (8)	
As1B	0.47520 (4)	0.605726 (14)	0.38588 (2)	0.01345 (11)	
As2B	0.60936 (4)	0.625216 (14)	0.53966 (2)	0.01376 (11)	
P1B	−0.00699 (10)	0.63514 (4)	0.38705 (6)	0.0135 (3)	
O1B	0.4252 (3)	0.70809 (10)	0.43994 (18)	0.0231 (9)	
O2B	0.4427 (3)	0.68995 (11)	0.6308 (2)	0.0325 (10)	
O3B	0.4406 (3)	0.56760 (11)	0.59609 (19)	0.0288 (10)	
O4B	0.3627 (4)	0.53067 (11)	0.4695 (2)	0.0338 (10)	
O5B	0.1563 (3)	0.56302 (11)	0.27274 (19)	0.0287 (9)	
O6B	0.2145 (3)	0.67645 (10)	0.31295 (18)	0.0206 (8)	
O7B	0.1899 (3)	0.72067 (10)	0.42444 (19)	0.0235 (9)	
O8B	0.1584 (3)	0.66850 (10)	0.57293 (19)	0.0258 (9)	
O9B	0.1874 (3)	0.55697 (10)	0.50761 (18)	0.0217 (8)	
C1B	0.4941 (4)	0.56427 (14)	0.3373 (2)	0.0156 (11)	
C2B	0.4412 (4)	0.53019 (14)	0.3351 (3)	0.0199 (12)	
H8	0.3959	0.5281	0.3552	0.024*	
C3B	0.4553 (4)	0.49962 (14)	0.3036 (3)	0.0199 (12)	
H9	0.4195	0.4771	0.3025	0.024*	
C4B	0.5229 (4)	0.50231 (15)	0.2733 (3)	0.0221 (12)	
H10	0.5330	0.4816	0.2521	0.027*	
C5B	0.5748 (4)	0.53597 (15)	0.2749 (3)	0.0229 (12)	
H11	0.6195	0.5379	0.2544	0.027*	
C6B	0.5612 (4)	0.56703 (14)	0.3067 (3)	0.0185 (11)	
H12	0.5968	0.5895	0.3076	0.022*	
C7B	0.5030 (4)	0.64876 (13)	0.3448 (2)	0.0141 (10)	
C8B	0.4311 (4)	0.65480 (15)	0.2796 (3)	0.0217 (12)	
H13	0.3721	0.6383	0.2576	0.026*	
C9B	0.4457 (5)	0.68492 (15)	0.2470 (3)	0.0249 (13)	
H14	0.3972	0.6886	0.2034	0.030*	
C10B	0.5342 (5)	0.70981 (15)	0.2803 (3)	0.0279 (14)	
H10B	0.5454	0.7300	0.2586	0.033*	
C11B	0.6051 (5)	0.70449 (15)	0.3454 (3)	0.0299 (14)	
H11B	0.6630	0.7214	0.3676	0.036*	
C12B	0.5899 (4)	0.67398 (14)	0.3777 (3)	0.0203 (12)	
H12B	0.6379	0.6704	0.4215	0.024*	
C13B	0.6145 (4)	0.60009 (14)	0.4663 (2)	0.0157 (11)	
H13C	0.6757	0.6105	0.4604	0.019*	
H13D	0.6294	0.5736	0.4760	0.019*	
C14B	0.7160 (4)	0.66613 (13)	0.5608 (2)	0.0143 (10)	
C15B	0.8239 (4)	0.65883 (17)	0.5704 (3)	0.0290 (14)	
H15B	0.8446	0.6345	0.5663	0.035*	
C16B	0.9001 (5)	0.68764 (16)	0.5862 (3)	0.0333 (15)	
H16B	0.9715	0.6828	0.5912	0.040*	
C17B	0.8714 (5)	0.72387 (16)	0.5945 (3)	0.0262 (13)	
H17B	0.9232	0.7433	0.6052	0.031*	
C18B	0.7661 (4)	0.73080 (14)	0.5868 (3)	0.0190 (11)	
H18B	0.7466	0.7550	0.5928	0.023*	
C19B	0.6876 (4)	0.70188 (14)	0.5702 (3)	0.0183 (11)	
H19B	0.6165	0.7068	0.5655	0.022*	
C20B	0.6949 (4)	0.59094 (15)	0.6106 (2)	0.0172 (11)	
C21B	0.7132 (5)	0.55357 (14)	0.6023 (3)	0.0232 (12)	
H21B	0.6862	0.5438	0.5609	0.028*	
C22B	0.7715 (5)	0.53071 (16)	0.6554 (3)	0.0273 (14)	
H22B	0.7833	0.5056	0.6492	0.033*	
C23B	0.8123 (5)	0.54455 (17)	0.7169 (3)	0.0295 (14)	
H23B	0.8514	0.5291	0.7525	0.035*	
C24B	0.7941 (5)	0.58221 (17)	0.7253 (3)	0.0321 (15)	
H24B	0.8215	0.5919	0.7668	0.038*	
C25B	0.7365 (5)	0.60513 (15)	0.6732 (3)	0.0272 (13)	
H25B	0.7250	0.6302	0.6796	0.033*	
C26B	−0.0677 (4)	0.59789 (14)	0.3264 (2)	0.0148 (10)	
C27B	−0.1726 (4)	0.60277 (16)	0.2732 (3)	0.0217 (12)	
H27B	−0.2098	0.6256	0.2672	0.026*	
C28B	−0.2215 (4)	0.57395 (15)	0.2294 (3)	0.0218 (12)	
H28B	−0.2916	0.5774	0.1942	0.026*	
C29B	−0.1667 (5)	0.54020 (16)	0.2378 (3)	0.0243 (13)	
H29B	−0.1995	0.5210	0.2079	0.029*	
C30B	−0.0630 (4)	0.53481 (15)	0.2905 (3)	0.0220 (12)	
H30B	−0.0264	0.5119	0.2965	0.026*	
C31B	−0.0134 (4)	0.56385 (14)	0.3346 (3)	0.0183 (11)	
H31B	0.0567	0.5603	0.3699	0.022*	
C32B	−0.0570 (4)	0.67746 (14)	0.3375 (2)	0.0171 (11)	
C33B	−0.0476 (4)	0.68106 (15)	0.2808 (3)	0.0191 (11)	
H33B	−0.0194	0.6611	0.2671	0.023*	
C34B	−0.0793 (4)	0.71365 (14)	0.2446 (3)	0.0216 (12)	
H34B	−0.0728	0.7155	0.2068	0.026*	
C35B	−0.1208 (5)	0.74337 (15)	0.2649 (3)	0.0269 (14)	
H35B	−0.1425	0.7653	0.2406	0.032*	
C36B	−0.1305 (5)	0.74073 (15)	0.3214 (3)	0.0254 (13)	
H36B	−0.1584	0.7609	0.3349	0.030*	
C37B	−0.0985 (4)	0.70808 (14)	0.3577 (3)	0.0213 (12)	
H37B	−0.1047	0.7064	0.3957	0.026*	
C38B	−0.1007 (4)	0.63267 (14)	0.4252 (3)	0.0179 (11)	
H38C	−0.0812	0.6529	0.4564	0.021*	
H38D	−0.1769	0.6370	0.3921	0.021*	
C39B	−0.0973 (4)	0.59617 (13)	0.4590 (2)	0.0137 (10)	
C40B	−0.0375 (4)	0.59257 (14)	0.5254 (3)	0.0205 (12)	
H40B	0.0064	0.6125	0.5499	0.025*	
C41B	−0.0420 (5)	0.55975 (15)	0.5562 (3)	0.0257 (13)	
H41B	−0.0015	0.5580	0.6009	0.031*	
C42B	−0.1057 (5)	0.52972 (16)	0.5211 (3)	0.0296 (14)	
H42B	−0.1084	0.5078	0.5420	0.036*	
C43B	−0.1655 (5)	0.53243 (15)	0.4546 (3)	0.0281 (14)	
H43B	−0.2090	0.5123	0.4306	0.034*	
C44B	−0.1606 (4)	0.56521 (15)	0.4236 (3)	0.0231 (12)	
H44B	−0.1997	0.5666	0.3787	0.028*	
C45B	0.4183 (4)	0.68217 (15)	0.4670 (3)	0.0171 (11)	
C46B	0.4380 (4)	0.67100 (15)	0.5898 (3)	0.0194 (12)	
C47B	0.4260 (4)	0.59422 (16)	0.5656 (3)	0.0207 (12)	
C48B	0.3413 (4)	0.55982 (15)	0.4451 (3)	0.0205 (12)	
C49B	0.2097 (4)	0.57950 (14)	0.3192 (3)	0.0196 (12)	
C50B	0.2496 (4)	0.65152 (15)	0.3488 (3)	0.0169 (11)	
C51B	0.1914 (4)	0.68920 (15)	0.4352 (3)	0.0174 (11)	
C52B	0.1674 (4)	0.65675 (14)	0.5298 (3)	0.0167 (11)	
C53B	0.1895 (4)	0.58647 (15)	0.4877 (2)	0.0167 (11)	

### Atomic displacement parameters (Å^2^)

**Table:**

	*U*^11^	*U*^22^	*U*^33^	*U*^12^	*U*^13^	*U*^23^
Ru1A	0.01086 (18)	0.0139 (2)	0.0140 (2)	−0.00043 (15)	0.00720 (16)	−0.00093 (16)
Ru2A	0.01012 (18)	0.0146 (2)	0.0134 (2)	−0.00005 (15)	0.00555 (16)	−0.00049 (16)
Ru3A	0.00996 (17)	0.01185 (19)	0.0137 (2)	0.00005 (15)	0.00659 (16)	−0.00070 (16)
As1A	0.0117 (2)	0.0154 (3)	0.0143 (3)	−0.00028 (19)	0.0076 (2)	−0.0005 (2)
As2A	0.0106 (2)	0.0142 (3)	0.0142 (3)	−0.00051 (19)	0.0059 (2)	−0.0009 (2)
P1A	0.0112 (6)	0.0133 (6)	0.0154 (7)	0.0002 (5)	0.0062 (5)	0.0008 (5)
O1A	0.038 (2)	0.022 (2)	0.036 (3)	0.0070 (18)	0.028 (2)	0.0072 (18)
O2A	0.031 (2)	0.038 (2)	0.026 (2)	−0.0107 (19)	0.016 (2)	−0.0140 (19)
O3A	0.023 (2)	0.020 (2)	0.025 (2)	0.0014 (16)	0.0112 (18)	0.0045 (17)
O4A	0.023 (2)	0.023 (2)	0.021 (2)	−0.0037 (16)	0.0071 (17)	0.0047 (17)
O5A	0.030 (2)	0.039 (2)	0.021 (2)	0.0047 (19)	0.0100 (19)	−0.0093 (19)
O6A	0.021 (2)	0.028 (2)	0.030 (2)	−0.0028 (17)	0.0085 (18)	0.0119 (19)
O7A	0.0189 (18)	0.0174 (19)	0.023 (2)	−0.0007 (15)	0.0141 (17)	0.0001 (16)
O8A	0.034 (2)	0.023 (2)	0.032 (2)	−0.0047 (17)	0.024 (2)	−0.0072 (18)
O9A	0.026 (2)	0.0147 (19)	0.029 (2)	0.0000 (16)	0.0147 (18)	0.0019 (16)
C1A	0.018 (2)	0.015 (3)	0.015 (3)	0.003 (2)	0.006 (2)	0.005 (2)
C2A	0.019 (3)	0.018 (3)	0.018 (3)	0.005 (2)	0.011 (2)	0.001 (2)
C3A	0.029 (3)	0.015 (3)	0.020 (3)	−0.004 (2)	0.013 (2)	−0.004 (2)
C4A	0.024 (3)	0.027 (3)	0.016 (3)	0.005 (2)	0.009 (2)	−0.005 (2)
C5A	0.020 (3)	0.035 (3)	0.022 (3)	0.002 (2)	0.015 (2)	−0.005 (3)
C6A	0.014 (3)	0.032 (3)	0.024 (3)	0.000 (2)	0.010 (2)	−0.003 (3)
C7A	0.012 (2)	0.016 (3)	0.026 (3)	−0.001 (2)	0.011 (2)	0.001 (2)
C8A	0.018 (3)	0.018 (3)	0.026 (3)	0.001 (2)	0.011 (2)	0.000 (2)
C9A	0.032 (3)	0.032 (3)	0.028 (3)	0.006 (3)	0.020 (3)	0.004 (3)
C10A	0.038 (3)	0.021 (3)	0.039 (4)	0.003 (3)	0.029 (3)	0.006 (3)
C11A	0.031 (3)	0.022 (3)	0.036 (4)	−0.009 (2)	0.023 (3)	−0.003 (3)
C12A	0.022 (3)	0.022 (3)	0.023 (3)	−0.001 (2)	0.013 (2)	−0.003 (2)
C13A	0.014 (2)	0.012 (2)	0.017 (3)	0.0021 (19)	0.005 (2)	−0.001 (2)
C14A	0.012 (2)	0.019 (3)	0.018 (3)	0.002 (2)	0.006 (2)	0.000 (2)
C15A	0.015 (2)	0.017 (3)	0.015 (3)	−0.002 (2)	0.008 (2)	−0.002 (2)
C16A	0.019 (3)	0.015 (3)	0.020 (3)	0.002 (2)	0.007 (2)	0.001 (2)
C17A	0.022 (3)	0.022 (3)	0.023 (3)	−0.007 (2)	0.011 (2)	−0.002 (2)
C18A	0.024 (3)	0.027 (3)	0.044 (4)	−0.005 (2)	0.026 (3)	−0.007 (3)
C19A	0.018 (3)	0.016 (3)	0.049 (4)	0.001 (2)	0.021 (3)	−0.005 (3)
C20A	0.012 (2)	0.020 (3)	0.017 (3)	−0.003 (2)	0.005 (2)	0.003 (2)
C21A	0.023 (3)	0.026 (3)	0.014 (3)	0.005 (2)	0.005 (2)	−0.006 (2)
C22A	0.031 (3)	0.021 (3)	0.030 (4)	0.009 (2)	0.007 (3)	0.004 (3)
C23A	0.024 (3)	0.038 (4)	0.019 (3)	−0.003 (3)	0.004 (3)	0.008 (3)
C24A	0.023 (3)	0.037 (3)	0.021 (3)	−0.004 (3)	0.010 (3)	0.003 (3)
C25A	0.022 (3)	0.032 (3)	0.020 (3)	−0.001 (2)	0.011 (2)	−0.001 (3)
C26A	0.016 (2)	0.015 (2)	0.015 (3)	−0.0058 (19)	0.011 (2)	−0.002 (2)
C27A	0.015 (2)	0.026 (3)	0.020 (3)	−0.003 (2)	0.010 (2)	0.001 (2)
C28A	0.019 (3)	0.026 (3)	0.021 (3)	−0.007 (2)	0.006 (2)	0.003 (2)
C29A	0.025 (3)	0.023 (3)	0.021 (3)	−0.010 (2)	0.014 (2)	−0.006 (2)
C30A	0.025 (3)	0.014 (3)	0.028 (3)	−0.004 (2)	0.014 (3)	−0.005 (2)
C31A	0.016 (2)	0.018 (3)	0.016 (3)	−0.006 (2)	0.008 (2)	−0.002 (2)
C32A	0.011 (2)	0.014 (3)	0.022 (3)	−0.0026 (19)	0.006 (2)	−0.003 (2)
C33A	0.025 (3)	0.017 (3)	0.029 (3)	0.003 (2)	0.014 (3)	0.002 (2)
C34A	0.030 (3)	0.019 (3)	0.037 (4)	0.006 (2)	0.014 (3)	0.000 (3)
C35A	0.027 (3)	0.019 (3)	0.030 (3)	0.002 (2)	0.005 (3)	0.011 (3)
C36A	0.025 (3)	0.022 (3)	0.018 (3)	−0.003 (2)	0.007 (2)	0.005 (2)
C37A	0.019 (3)	0.018 (3)	0.024 (3)	0.000 (2)	0.009 (2)	−0.002 (2)
C38A	0.012 (2)	0.017 (3)	0.021 (3)	0.0002 (19)	0.006 (2)	−0.001 (2)
C39A	0.015 (2)	0.022 (3)	0.022 (3)	0.001 (2)	0.014 (2)	0.000 (2)
C40A	0.019 (3)	0.027 (3)	0.021 (3)	−0.008 (2)	0.011 (2)	−0.001 (2)
C41A	0.029 (3)	0.020 (3)	0.040 (4)	−0.005 (2)	0.024 (3)	0.002 (3)
C42A	0.029 (3)	0.030 (3)	0.030 (3)	0.001 (3)	0.021 (3)	0.008 (3)
C43A	0.028 (3)	0.038 (4)	0.023 (3)	0.004 (3)	0.020 (3)	0.004 (3)
C44A	0.013 (2)	0.027 (3)	0.023 (3)	0.001 (2)	0.011 (2)	−0.002 (2)
C45A	0.020 (3)	0.022 (3)	0.025 (3)	0.001 (2)	0.019 (2)	−0.005 (2)
C46A	0.022 (3)	0.022 (3)	0.019 (3)	0.001 (2)	0.017 (2)	−0.001 (2)
C47A	0.014 (2)	0.023 (3)	0.015 (3)	−0.003 (2)	0.007 (2)	−0.004 (2)
C48A	0.009 (2)	0.028 (3)	0.015 (3)	0.000 (2)	0.001 (2)	−0.005 (2)
C49A	0.010 (2)	0.026 (3)	0.022 (3)	0.000 (2)	0.005 (2)	0.001 (2)
C50A	0.012 (2)	0.022 (3)	0.023 (3)	−0.002 (2)	0.006 (2)	−0.003 (2)
C51A	0.011 (2)	0.020 (3)	0.012 (3)	−0.001 (2)	0.007 (2)	−0.003 (2)
C52A	0.017 (2)	0.011 (2)	0.018 (3)	0.0007 (19)	0.007 (2)	0.001 (2)
C53A	0.011 (2)	0.026 (3)	0.014 (3)	0.003 (2)	0.008 (2)	−0.003 (2)
Ru1B	0.01150 (18)	0.0155 (2)	0.0125 (2)	0.00016 (16)	0.00559 (16)	−0.00002 (17)
Ru2B	0.01322 (19)	0.0133 (2)	0.0143 (2)	−0.00050 (15)	0.00783 (17)	−0.00109 (16)
Ru3B	0.01116 (18)	0.01171 (19)	0.0140 (2)	−0.00040 (15)	0.00686 (16)	−0.00116 (16)
As1B	0.0138 (2)	0.0140 (3)	0.0142 (3)	0.0007 (2)	0.0078 (2)	−0.0001 (2)
As2B	0.0118 (2)	0.0154 (3)	0.0140 (3)	0.00046 (19)	0.0059 (2)	0.0000 (2)
P1B	0.0123 (6)	0.0131 (6)	0.0159 (7)	−0.0003 (5)	0.0071 (5)	−0.0006 (5)
O1B	0.021 (2)	0.020 (2)	0.024 (2)	−0.0029 (16)	0.0076 (17)	0.0048 (17)
O2B	0.040 (3)	0.036 (2)	0.026 (2)	−0.005 (2)	0.019 (2)	−0.011 (2)
O3B	0.026 (2)	0.027 (2)	0.033 (2)	0.0011 (17)	0.0131 (19)	0.0128 (19)
O4B	0.061 (3)	0.018 (2)	0.034 (3)	0.007 (2)	0.031 (2)	0.0053 (19)
O5B	0.024 (2)	0.037 (2)	0.026 (2)	−0.0103 (18)	0.0122 (19)	−0.0140 (19)
O6B	0.022 (2)	0.0193 (19)	0.023 (2)	0.0029 (16)	0.0128 (17)	0.0023 (17)
O7B	0.025 (2)	0.016 (2)	0.034 (2)	−0.0003 (16)	0.0175 (19)	0.0019 (17)
O8B	0.036 (2)	0.024 (2)	0.025 (2)	−0.0056 (18)	0.0203 (19)	−0.0099 (18)
O9B	0.025 (2)	0.0172 (19)	0.022 (2)	−0.0045 (16)	0.0111 (17)	0.0009 (16)
C1B	0.017 (2)	0.016 (3)	0.012 (3)	−0.001 (2)	0.006 (2)	−0.003 (2)
C2B	0.023 (3)	0.021 (3)	0.021 (3)	0.003 (2)	0.015 (2)	0.002 (2)
C3B	0.026 (3)	0.012 (3)	0.022 (3)	0.002 (2)	0.011 (2)	0.002 (2)
C4B	0.023 (3)	0.025 (3)	0.020 (3)	0.005 (2)	0.011 (2)	−0.004 (2)
C5B	0.022 (3)	0.032 (3)	0.022 (3)	0.000 (2)	0.017 (3)	−0.006 (2)
C6B	0.019 (3)	0.017 (3)	0.022 (3)	−0.002 (2)	0.012 (2)	0.000 (2)
C7B	0.017 (2)	0.017 (3)	0.017 (3)	0.002 (2)	0.015 (2)	0.000 (2)
C8B	0.018 (3)	0.022 (3)	0.025 (3)	0.001 (2)	0.010 (2)	0.000 (2)
C9B	0.031 (3)	0.022 (3)	0.025 (3)	0.006 (2)	0.016 (3)	0.005 (2)
C10B	0.042 (4)	0.020 (3)	0.040 (4)	0.000 (3)	0.035 (3)	0.004 (3)
C11B	0.039 (4)	0.020 (3)	0.041 (4)	−0.008 (3)	0.027 (3)	−0.007 (3)
C12B	0.020 (3)	0.023 (3)	0.019 (3)	−0.006 (2)	0.011 (2)	−0.002 (2)
C13B	0.016 (2)	0.014 (2)	0.014 (3)	0.006 (2)	0.005 (2)	0.000 (2)
C14B	0.017 (2)	0.016 (3)	0.013 (3)	−0.005 (2)	0.010 (2)	−0.002 (2)
C15B	0.018 (3)	0.031 (3)	0.040 (4)	−0.003 (2)	0.016 (3)	−0.010 (3)
C16B	0.015 (3)	0.029 (3)	0.057 (4)	−0.005 (2)	0.018 (3)	−0.012 (3)
C17B	0.024 (3)	0.028 (3)	0.030 (3)	−0.008 (2)	0.016 (3)	−0.006 (3)
C18B	0.020 (3)	0.016 (3)	0.021 (3)	0.004 (2)	0.010 (2)	0.000 (2)
C19B	0.017 (3)	0.018 (3)	0.021 (3)	−0.003 (2)	0.009 (2)	−0.001 (2)
C20B	0.008 (2)	0.027 (3)	0.016 (3)	−0.002 (2)	0.005 (2)	0.004 (2)
C21B	0.029 (3)	0.018 (3)	0.021 (3)	0.002 (2)	0.010 (3)	0.003 (2)
C22B	0.028 (3)	0.021 (3)	0.029 (3)	0.001 (2)	0.010 (3)	0.010 (3)
C23B	0.021 (3)	0.035 (4)	0.024 (3)	−0.004 (3)	0.002 (3)	0.014 (3)
C24B	0.037 (4)	0.042 (4)	0.010 (3)	−0.006 (3)	0.004 (3)	−0.004 (3)
C25B	0.037 (3)	0.018 (3)	0.024 (3)	0.002 (2)	0.012 (3)	0.001 (2)
C26B	0.015 (2)	0.020 (3)	0.011 (2)	−0.003 (2)	0.008 (2)	0.000 (2)
C27B	0.016 (3)	0.028 (3)	0.021 (3)	−0.005 (2)	0.008 (2)	0.001 (2)
C28B	0.020 (3)	0.033 (3)	0.014 (3)	−0.010 (2)	0.008 (2)	−0.003 (2)
C29B	0.029 (3)	0.029 (3)	0.018 (3)	−0.014 (2)	0.014 (3)	−0.012 (2)
C30B	0.024 (3)	0.020 (3)	0.027 (3)	−0.003 (2)	0.016 (3)	−0.009 (2)
C31B	0.015 (2)	0.025 (3)	0.017 (3)	−0.004 (2)	0.010 (2)	−0.003 (2)
C32B	0.011 (2)	0.019 (3)	0.015 (3)	−0.003 (2)	0.000 (2)	0.001 (2)
C33B	0.014 (2)	0.021 (3)	0.021 (3)	−0.003 (2)	0.007 (2)	−0.001 (2)
C34B	0.021 (3)	0.022 (3)	0.019 (3)	−0.006 (2)	0.007 (2)	0.004 (2)
C35B	0.026 (3)	0.016 (3)	0.030 (3)	0.001 (2)	0.006 (3)	0.007 (2)
C36B	0.032 (3)	0.019 (3)	0.025 (3)	0.003 (2)	0.014 (3)	−0.001 (2)
C37B	0.024 (3)	0.019 (3)	0.021 (3)	0.002 (2)	0.011 (2)	0.001 (2)
C38B	0.017 (2)	0.017 (3)	0.027 (3)	0.000 (2)	0.016 (2)	−0.002 (2)
C39B	0.012 (2)	0.012 (2)	0.022 (3)	−0.0024 (19)	0.012 (2)	−0.003 (2)
C40B	0.020 (3)	0.015 (3)	0.032 (3)	−0.005 (2)	0.017 (3)	−0.001 (2)
C41B	0.031 (3)	0.028 (3)	0.022 (3)	0.001 (3)	0.017 (3)	0.002 (3)
C42B	0.042 (4)	0.018 (3)	0.035 (4)	−0.005 (3)	0.023 (3)	0.000 (3)
C43B	0.037 (3)	0.016 (3)	0.037 (4)	−0.011 (2)	0.022 (3)	−0.007 (3)
C44B	0.020 (3)	0.027 (3)	0.027 (3)	−0.003 (2)	0.014 (3)	−0.003 (3)
C45B	0.009 (2)	0.025 (3)	0.016 (3)	0.001 (2)	0.005 (2)	−0.005 (2)
C46B	0.016 (3)	0.022 (3)	0.020 (3)	0.002 (2)	0.009 (2)	0.001 (2)
C47B	0.012 (2)	0.030 (3)	0.022 (3)	−0.001 (2)	0.010 (2)	−0.001 (3)
C48B	0.025 (3)	0.022 (3)	0.020 (3)	−0.004 (2)	0.016 (2)	−0.004 (2)
C49B	0.019 (3)	0.019 (3)	0.026 (3)	0.003 (2)	0.015 (2)	0.004 (2)
C50B	0.016 (2)	0.021 (3)	0.016 (3)	−0.003 (2)	0.010 (2)	−0.006 (2)
C51B	0.014 (2)	0.019 (3)	0.021 (3)	0.001 (2)	0.010 (2)	0.001 (2)
C52B	0.012 (2)	0.015 (3)	0.020 (3)	−0.0051 (19)	0.006 (2)	−0.005 (2)
C53B	0.013 (2)	0.026 (3)	0.014 (3)	−0.005 (2)	0.008 (2)	−0.008 (2)

### Geometric parameters (Å, °)

**Table:**

Ru1A—C46A	1.878 (6)	Ru1B—C46B	1.886 (6)
Ru1A—C47A	1.926 (5)	Ru1B—C45B	1.929 (6)
Ru1A—C45A	1.930 (6)	Ru1B—C47B	1.937 (6)
Ru1A—As1A	2.4240 (6)	Ru1B—As2B	2.4271 (6)
Ru1A—Ru2A	2.8679 (6)	Ru1B—Ru3B	2.8331 (5)
Ru1A—Ru3A	2.8855 (6)	Ru1B—Ru2B	2.8558 (6)
Ru2A—C49A	1.905 (6)	Ru2B—C49B	1.893 (6)
Ru2A—C48A	1.924 (6)	Ru2B—C50B	1.912 (5)
Ru2A—C50A	1.928 (6)	Ru2B—C48B	1.924 (6)
Ru2A—As2A	2.4261 (6)	Ru2B—As1B	2.4308 (6)
Ru2A—Ru3A	2.8395 (5)	Ru2B—Ru3B	2.8970 (6)
Ru3A—C52A	1.880 (6)	Ru3B—C52B	1.869 (6)
Ru3A—C51A	1.931 (5)	Ru3B—C53B	1.927 (5)
Ru3A—C53A	1.948 (5)	Ru3B—C51B	1.937 (5)
Ru3A—P1A	2.3414 (13)	Ru3B—P1B	2.3374 (13)
As1A—C7A	1.945 (5)	As1B—C7B	1.935 (5)
As1A—C1A	1.953 (5)	As1B—C1B	1.949 (5)
As1A—C13A	1.962 (5)	As1B—C13B	1.956 (5)
As2A—C20A	1.935 (5)	As2B—C14B	1.942 (5)
As2A—C14A	1.938 (5)	As2B—C20B	1.947 (5)
As2A—C13A	1.952 (5)	As2B—C13B	1.962 (5)
P1A—C32A	1.824 (5)	P1B—C32B	1.828 (5)
P1A—C26A	1.825 (5)	P1B—C26B	1.833 (5)
P1A—C38A	1.854 (5)	P1B—C38B	1.855 (5)
O1A—C45A	1.148 (6)	O1B—C45B	1.147 (6)
O2A—C46A	1.155 (6)	O2B—C46B	1.147 (6)
O3A—C47A	1.149 (6)	O3B—C47B	1.146 (6)
O4A—C48A	1.153 (6)	O4B—C48B	1.155 (6)
O5A—C49A	1.136 (6)	O5B—C49B	1.147 (6)
O6A—C50A	1.146 (6)	O6B—C50B	1.160 (6)
O7A—C51A	1.147 (6)	O7B—C51B	1.146 (6)
O8A—C52A	1.146 (6)	O8B—C52B	1.148 (6)
O9A—C53A	1.142 (6)	O9B—C53B	1.153 (6)
C1A—C2A	1.387 (7)	C1B—C6B	1.391 (7)
C1A—C6A	1.396 (7)	C1B—C2B	1.396 (7)
C2A—C3A	1.381 (7)	C2B—C3B	1.374 (7)
C2A—H1	0.9300	C2B—H8	0.9300
C3A—C4A	1.381 (7)	C3B—C4B	1.393 (7)
C3A—H2	0.9300	C3B—H9	0.9300
C4A—C5A	1.372 (8)	C4B—C5B	1.378 (7)
C4A—H3	0.9300	C4B—H10	0.9300
C5A—C6A	1.380 (7)	C5B—C6B	1.391 (7)
C5A—H4	0.9300	C5B—H11	0.9300
C6A—H5	0.9300	C6B—H12	0.9300
C7A—C8A	1.394 (7)	C7B—C8B	1.390 (7)
C7A—C12A	1.399 (7)	C7B—C12B	1.391 (7)
C8A—C9A	1.378 (7)	C8B—C9B	1.381 (7)
C8A—H6	0.9300	C8B—H13	0.9300
C9A—C10A	1.387 (8)	C9B—C10B	1.398 (8)
C9A—H7	0.9300	C9B—H14	0.9300
C10A—C11A	1.377 (8)	C10B—C11B	1.384 (8)
C10A—H10A	0.9300	C10B—H10B	0.9300
C11A—C12A	1.384 (7)	C11B—C12B	1.391 (7)
C11A—H11A	0.9300	C11B—H11B	0.9300
C12A—H12A	0.9300	C12B—H12B	0.9300
C13A—H13A	0.9700	C13B—H13C	0.9700
C13A—H13B	0.9700	C13B—H13D	0.9700
C14A—C19A	1.384 (7)	C14B—C19B	1.375 (7)
C14A—C15A	1.390 (7)	C14B—C15B	1.391 (7)
C15A—C16A	1.380 (7)	C15B—C16B	1.376 (7)
C15A—H15A	0.9300	C15B—H15B	0.9300
C16A—C17A	1.387 (7)	C16B—C17B	1.386 (8)
C16A—H16A	0.9300	C16B—H16B	0.9300
C17A—C18A	1.381 (7)	C17B—C18B	1.369 (7)
C17A—H17A	0.9300	C17B—H17B	0.9300
C18A—C19A	1.381 (7)	C18B—C19B	1.396 (7)
C18A—H18A	0.9300	C18B—H18B	0.9300
C19A—H19A	0.9300	C19B—H19B	0.9300
C20A—C21A	1.376 (7)	C20B—C21B	1.383 (7)
C20A—C25A	1.396 (7)	C20B—C25B	1.396 (7)
C21A—C22A	1.405 (7)	C21B—C22B	1.383 (7)
C21A—H21A	0.9300	C21B—H21B	0.9300
C22A—C23A	1.362 (8)	C22B—C23B	1.371 (8)
C22A—H22A	0.9300	C22B—H22B	0.9300
C23A—C24A	1.384 (8)	C23B—C24B	1.393 (8)
C23A—H23A	0.9300	C23B—H23B	0.9300
C24A—C25A	1.380 (8)	C24B—C25B	1.368 (8)
C24A—H24A	0.9300	C24B—H24B	0.9300
C25A—H25A	0.9300	C25B—H25B	0.9300
C26A—C31A	1.398 (7)	C26B—C31B	1.382 (7)
C26A—C27A	1.401 (7)	C26B—C27B	1.395 (7)
C27A—C28A	1.380 (7)	C27B—C28B	1.381 (7)
C27A—H27A	0.9300	C27B—H27B	0.9300
C28A—C29A	1.378 (7)	C28B—C29B	1.376 (8)
C28A—H28A	0.9300	C28B—H28B	0.9300
C29A—C30A	1.387 (7)	C29B—C30B	1.384 (7)
C29A—H29A	0.9300	C29B—H29B	0.9300
C30A—C31A	1.383 (7)	C30B—C31B	1.392 (7)
C30A—H30A	0.9300	C30B—H30B	0.9300
C31A—H31A	0.9300	C31B—H31B	0.9300
C32A—C37A	1.390 (7)	C32B—C33B	1.391 (7)
C32A—C33A	1.393 (7)	C32B—C37B	1.403 (7)
C33A—C34A	1.378 (7)	C33B—C34B	1.382 (7)
C33A—H33A	0.9300	C33B—H33B	0.9300
C34A—C35A	1.382 (8)	C34B—C35B	1.380 (8)
C34A—H34A	0.9300	C34B—H34B	0.9300
C35A—C36A	1.392 (8)	C35B—C36B	1.387 (8)
C35A—H35A	0.9300	C35B—H35B	0.9300
C36A—C37A	1.379 (7)	C36B—C37B	1.384 (7)
C36A—H36A	0.9300	C36B—H36B	0.9300
C37A—H37A	0.9300	C37B—H37B	0.9300
C38A—C39A	1.514 (7)	C38B—C39B	1.510 (7)
C38A—H38A	0.9700	C38B—H38C	0.9700
C38A—H38B	0.9700	C38B—H38D	0.9700
C39A—C44A	1.399 (7)	C39B—C40B	1.383 (7)
C39A—C40A	1.401 (7)	C39B—C44B	1.404 (7)
C40A—C41A	1.383 (7)	C40B—C41B	1.387 (7)
C40A—H40A	0.9300	C40B—H40B	0.9300
C41A—C42A	1.380 (8)	C41B—C42B	1.378 (8)
C41A—H41A	0.9300	C41B—H41B	0.9300
C42A—C43A	1.372 (8)	C42B—C43B	1.383 (8)
C42A—H42A	0.9300	C42B—H42B	0.9300
C43A—C44A	1.393 (7)	C43B—C44B	1.391 (7)
C43A—H43A	0.9300	C43B—H43B	0.9300
C44A—H44A	0.9300	C44B—H44B	0.9300
			
C46A—Ru1A—C47A	87.6 (2)	C46B—Ru1B—C45B	93.7 (2)
C46A—Ru1A—C45A	92.6 (2)	C46B—Ru1B—C47B	93.3 (2)
C47A—Ru1A—C45A	172.7 (2)	C45B—Ru1B—C47B	172.8 (2)
C46A—Ru1A—As1A	98.57 (16)	C46B—Ru1B—As2B	105.38 (16)
C47A—Ru1A—As1A	94.04 (15)	C45B—Ru1B—As2B	89.77 (14)
C45A—Ru1A—As1A	93.12 (15)	C47B—Ru1B—As2B	86.61 (15)
C46A—Ru1A—Ru2A	170.58 (16)	C46B—Ru1B—Ru3B	96.06 (15)
C47A—Ru1A—Ru2A	95.09 (15)	C45B—Ru1B—Ru3B	91.56 (14)
C45A—Ru1A—Ru2A	83.59 (16)	C47B—Ru1B—Ru3B	89.53 (15)
As1A—Ru1A—Ru2A	90.257 (19)	As2B—Ru1B—Ru3B	158.39 (2)
C46A—Ru1A—Ru3A	112.48 (16)	C46B—Ru1B—Ru2B	156.29 (15)
C47A—Ru1A—Ru3A	82.32 (15)	C45B—Ru1B—Ru2B	81.09 (15)
C45A—Ru1A—Ru3A	90.89 (15)	C47B—Ru1B—Ru2B	93.21 (16)
As1A—Ru1A—Ru3A	148.47 (2)	As2B—Ru1B—Ru2B	97.74 (2)
Ru2A—Ru1A—Ru3A	59.145 (13)	Ru3B—Ru1B—Ru2B	61.225 (14)
C49A—Ru2A—C48A	93.1 (2)	C49B—Ru2B—C50B	88.1 (2)
C49A—Ru2A—C50A	91.9 (2)	C49B—Ru2B—C48B	91.2 (2)
C48A—Ru2A—C50A	174.9 (2)	C50B—Ru2B—C48B	172.7 (2)
C49A—Ru2A—As2A	104.90 (15)	C49B—Ru2B—As1B	99.08 (16)
C48A—Ru2A—As2A	90.66 (15)	C50B—Ru2B—As1B	94.01 (15)
C50A—Ru2A—As2A	88.01 (15)	C48B—Ru2B—As1B	93.29 (16)
C49A—Ru2A—Ru3A	98.08 (15)	C49B—Ru2B—Ru1B	170.15 (16)
C48A—Ru2A—Ru3A	93.17 (15)	C50B—Ru2B—Ru1B	95.78 (15)
C50A—Ru2A—Ru3A	86.14 (15)	C48B—Ru2B—Ru1B	83.77 (16)
As2A—Ru2A—Ru3A	156.46 (2)	As1B—Ru2B—Ru1B	89.711 (19)
C49A—Ru2A—Ru1A	157.34 (15)	C49B—Ru2B—Ru3B	113.21 (16)
C48A—Ru2A—Ru1A	81.00 (15)	C50B—Ru2B—Ru3B	78.84 (15)
C50A—Ru2A—Ru1A	94.32 (16)	C48B—Ru2B—Ru3B	94.76 (16)
As2A—Ru2A—Ru1A	97.08 (2)	As1B—Ru2B—Ru3B	146.46 (2)
Ru3A—Ru2A—Ru1A	60.736 (14)	Ru1B—Ru2B—Ru3B	59.003 (14)
C52A—Ru3A—C51A	90.4 (2)	C52B—Ru3B—C53B	93.0 (2)
C52A—Ru3A—C53A	89.8 (2)	C52B—Ru3B—C51B	87.9 (2)
C51A—Ru3A—C53A	176.3 (2)	C53B—Ru3B—C51B	175.2 (2)
C52A—Ru3A—P1A	99.80 (15)	C52B—Ru3B—P1B	96.66 (15)
C51A—Ru3A—P1A	93.01 (14)	C53B—Ru3B—P1B	93.83 (15)
C53A—Ru3A—P1A	90.56 (15)	C51B—Ru3B—P1B	90.74 (15)
C52A—Ru3A—Ru2A	95.69 (15)	C52B—Ru3B—Ru1B	96.81 (15)
C51A—Ru3A—Ru2A	92.53 (14)	C53B—Ru3B—Ru1B	90.14 (14)
C53A—Ru3A—Ru2A	83.81 (14)	C51B—Ru3B—Ru1B	85.09 (15)
P1A—Ru3A—Ru2A	163.50 (4)	P1B—Ru3B—Ru1B	165.73 (4)
C52A—Ru3A—Ru1A	155.00 (15)	C52B—Ru3B—Ru2B	155.75 (15)
C51A—Ru3A—Ru1A	84.96 (14)	C53B—Ru3B—Ru2B	81.65 (15)
C53A—Ru3A—Ru1A	93.33 (15)	C51B—Ru3B—Ru2B	95.63 (15)
P1A—Ru3A—Ru1A	104.95 (4)	P1B—Ru3B—Ru2B	107.25 (4)
Ru2A—Ru3A—Ru1A	60.119 (14)	Ru1B—Ru3B—Ru2B	59.772 (14)
C7A—As1A—C1A	101.7 (2)	C7B—As1B—C1B	101.7 (2)
C7A—As1A—C13A	104.6 (2)	C7B—As1B—C13B	103.7 (2)
C1A—As1A—C13A	97.5 (2)	C1B—As1B—C13B	98.9 (2)
C7A—As1A—Ru1A	117.66 (14)	C7B—As1B—Ru2B	117.40 (14)
C1A—As1A—Ru1A	117.37 (15)	C1B—As1B—Ru2B	116.30 (15)
C13A—As1A—Ru1A	115.14 (15)	C13B—As1B—Ru2B	116.16 (15)
C20A—As2A—C14A	98.3 (2)	C14B—As2B—C20B	100.2 (2)
C20A—As2A—C13A	102.1 (2)	C14B—As2B—C13B	104.0 (2)
C14A—As2A—C13A	103.2 (2)	C20B—As2B—C13B	102.1 (2)
C20A—As2A—Ru2A	116.95 (15)	C14B—As2B—Ru1B	118.05 (15)
C14A—As2A—Ru2A	120.04 (15)	C20B—As2B—Ru1B	117.07 (15)
C13A—As2A—Ru2A	113.48 (14)	C13B—As2B—Ru1B	113.18 (14)
C32A—P1A—C26A	103.3 (2)	C32B—P1B—C26B	101.9 (2)
C32A—P1A—C38A	102.4 (2)	C32B—P1B—C38B	102.1 (2)
C26A—P1A—C38A	100.1 (2)	C26B—P1B—C38B	100.2 (2)
C32A—P1A—Ru3A	113.06 (16)	C32B—P1B—Ru3B	112.77 (16)
C26A—P1A—Ru3A	120.81 (17)	C26B—P1B—Ru3B	122.61 (17)
C38A—P1A—Ru3A	114.80 (17)	C38B—P1B—Ru3B	114.57 (18)
C2A—C1A—C6A	118.6 (5)	C6B—C1B—C2B	119.0 (5)
C2A—C1A—As1A	118.4 (4)	C6B—C1B—As1B	122.5 (4)
C6A—C1A—As1A	122.9 (4)	C2B—C1B—As1B	118.5 (4)
C3A—C2A—C1A	121.4 (5)	C3B—C2B—C1B	120.9 (5)
C3A—C2A—H1	119.3	C3B—C2B—H8	119.5
C1A—C2A—H1	119.3	C1B—C2B—H8	119.5
C4A—C3A—C2A	119.2 (5)	C2B—C3B—C4B	120.1 (5)
C4A—C3A—H2	120.4	C2B—C3B—H9	119.9
C2A—C3A—H2	120.4	C4B—C3B—H9	119.9
C5A—C4A—C3A	120.2 (5)	C5B—C4B—C3B	119.2 (5)
C5A—C4A—H3	119.9	C5B—C4B—H10	120.4
C3A—C4A—H3	119.9	C3B—C4B—H10	120.4
C4A—C5A—C6A	120.8 (5)	C4B—C5B—C6B	121.0 (5)
C4A—C5A—H4	119.6	C4B—C5B—H11	119.5
C6A—C5A—H4	119.6	C6B—C5B—H11	119.5
C5A—C6A—C1A	119.8 (5)	C5B—C6B—C1B	119.7 (5)
C5A—C6A—H5	120.1	C5B—C6B—H12	120.1
C1A—C6A—H5	120.1	C1B—C6B—H12	120.1
C8A—C7A—C12A	120.0 (5)	C8B—C7B—C12B	119.2 (5)
C8A—C7A—As1A	117.6 (4)	C8B—C7B—As1B	117.9 (4)
C12A—C7A—As1A	122.4 (4)	C12B—C7B—As1B	122.9 (4)
C9A—C8A—C7A	119.9 (5)	C9B—C8B—C7B	121.2 (5)
C9A—C8A—H6	120.0	C9B—C8B—H13	119.4
C7A—C8A—H6	120.0	C7B—C8B—H13	119.4
C8A—C9A—C10A	120.0 (6)	C8B—C9B—C10B	119.3 (5)
C8A—C9A—H7	120.0	C8B—C9B—H14	120.4
C10A—C9A—H7	120.0	C10B—C9B—H14	120.4
C11A—C10A—C9A	120.2 (5)	C11B—C10B—C9B	120.1 (5)
C11A—C10A—H10A	119.9	C11B—C10B—H10B	120.0
C9A—C10A—H10A	119.9	C9B—C10B—H10B	120.0
C10A—C11A—C12A	120.7 (5)	C10B—C11B—C12B	120.2 (5)
C10A—C11A—H11A	119.6	C10B—C11B—H11B	119.9
C12A—C11A—H11A	119.6	C12B—C11B—H11B	119.9
C11A—C12A—C7A	119.1 (5)	C11B—C12B—C7B	120.1 (5)
C11A—C12A—H12A	120.4	C11B—C12B—H12B	120.0
C7A—C12A—H12A	120.4	C7B—C12B—H12B	120.0
As2A—C13A—As1A	112.3 (2)	As1B—C13B—As2B	112.6 (2)
As2A—C13A—H13A	109.1	As1B—C13B—H13C	109.1
As1A—C13A—H13A	109.1	As2B—C13B—H13C	109.1
As2A—C13A—H13B	109.1	As1B—C13B—H13D	109.1
As1A—C13A—H13B	109.1	As2B—C13B—H13D	109.1
H13A—C13A—H13B	107.9	H13C—C13B—H13D	107.8
C19A—C14A—C15A	119.5 (5)	C19B—C14B—C15B	119.6 (5)
C19A—C14A—As2A	119.9 (4)	C19B—C14B—As2B	120.5 (4)
C15A—C14A—As2A	120.5 (4)	C15B—C14B—As2B	119.8 (4)
C16A—C15A—C14A	120.1 (5)	C16B—C15B—C14B	120.0 (5)
C16A—C15A—H15A	120.0	C16B—C15B—H15B	120.0
C14A—C15A—H15A	120.0	C14B—C15B—H15B	120.0
C15A—C16A—C17A	120.0 (5)	C15B—C16B—C17B	120.6 (5)
C15A—C16A—H16A	120.0	C15B—C16B—H16B	119.7
C17A—C16A—H16A	120.0	C17B—C16B—H16B	119.7
C18A—C17A—C16A	120.0 (5)	C18B—C17B—C16B	119.3 (5)
C18A—C17A—H17A	120.0	C18B—C17B—H17B	120.3
C16A—C17A—H17A	120.0	C16B—C17B—H17B	120.3
C19A—C18A—C17A	119.9 (5)	C17B—C18B—C19B	120.6 (5)
C19A—C18A—H18A	120.0	C17B—C18B—H18B	119.7
C17A—C18A—H18A	120.0	C19B—C18B—H18B	119.7
C18A—C19A—C14A	120.5 (5)	C14B—C19B—C18B	119.8 (5)
C18A—C19A—H19A	119.8	C14B—C19B—H19B	120.1
C14A—C19A—H19A	119.8	C18B—C19B—H19B	120.1
C21A—C20A—C25A	119.1 (5)	C21B—C20B—C25B	118.9 (5)
C21A—C20A—As2A	124.1 (4)	C21B—C20B—As2B	123.9 (4)
C25A—C20A—As2A	116.6 (4)	C25B—C20B—As2B	117.2 (4)
C20A—C21A—C22A	119.9 (5)	C22B—C21B—C20B	120.4 (5)
C20A—C21A—H21A	120.0	C22B—C21B—H21B	119.8
C22A—C21A—H21A	120.0	C20B—C21B—H21B	119.8
C23A—C22A—C21A	120.6 (5)	C23B—C22B—C21B	120.9 (5)
C23A—C22A—H22A	119.7	C23B—C22B—H22B	119.6
C21A—C22A—H22A	119.7	C21B—C22B—H22B	119.6
C22A—C23A—C24A	119.5 (5)	C22B—C23B—C24B	118.8 (5)
C22A—C23A—H23A	120.2	C22B—C23B—H23B	120.6
C24A—C23A—H23A	120.2	C24B—C23B—H23B	120.6
C25A—C24A—C23A	120.5 (6)	C25B—C24B—C23B	120.9 (5)
C25A—C24A—H24A	119.7	C25B—C24B—H24B	119.5
C23A—C24A—H24A	119.7	C23B—C24B—H24B	119.5
C24A—C25A—C20A	120.2 (5)	C24B—C25B—C20B	120.2 (5)
C24A—C25A—H25A	119.9	C24B—C25B—H25B	119.9
C20A—C25A—H25A	119.9	C20B—C25B—H25B	119.9
C31A—C26A—C27A	117.5 (5)	C31B—C26B—C27B	119.1 (5)
C31A—C26A—P1A	121.0 (4)	C31B—C26B—P1B	120.6 (4)
C27A—C26A—P1A	121.1 (4)	C27B—C26B—P1B	120.2 (4)
C28A—C27A—C26A	120.9 (5)	C28B—C27B—C26B	120.4 (5)
C28A—C27A—H27A	119.6	C28B—C27B—H27B	119.8
C26A—C27A—H27A	119.6	C26B—C27B—H27B	119.8
C29A—C28A—C27A	120.6 (5)	C29B—C28B—C27B	120.2 (5)
C29A—C28A—H28A	119.7	C29B—C28B—H28B	119.9
C27A—C28A—H28A	119.7	C27B—C28B—H28B	119.9
C28A—C29A—C30A	119.8 (5)	C28B—C29B—C30B	120.1 (5)
C28A—C29A—H29A	120.1	C28B—C29B—H29B	119.9
C30A—C29A—H29A	120.1	C30B—C29B—H29B	119.9
C31A—C30A—C29A	119.5 (5)	C29B—C30B—C31B	119.8 (5)
C31A—C30A—H30A	120.2	C29B—C30B—H30B	120.1
C29A—C30A—H30A	120.2	C31B—C30B—H30B	120.1
C30A—C31A—C26A	121.6 (5)	C26B—C31B—C30B	120.4 (5)
C30A—C31A—H31A	119.2	C26B—C31B—H31B	119.8
C26A—C31A—H31A	119.2	C30B—C31B—H31B	119.8
C37A—C32A—C33A	118.0 (5)	C33B—C32B—C37B	118.3 (5)
C37A—C32A—P1A	119.7 (4)	C33B—C32B—P1B	120.6 (4)
C33A—C32A—P1A	121.9 (4)	C37B—C32B—P1B	121.0 (4)
C34A—C33A—C32A	120.9 (6)	C34B—C33B—C32B	121.4 (5)
C34A—C33A—H33A	119.6	C34B—C33B—H33B	119.3
C32A—C33A—H33A	119.6	C32B—C33B—H33B	119.3
C33A—C34A—C35A	120.3 (5)	C35B—C34B—C33B	119.5 (5)
C33A—C34A—H34A	119.8	C35B—C34B—H34B	120.2
C35A—C34A—H34A	119.8	C33B—C34B—H34B	120.2
C34A—C35A—C36A	119.8 (5)	C34B—C35B—C36B	120.5 (5)
C34A—C35A—H35A	120.1	C34B—C35B—H35B	119.8
C36A—C35A—H35A	120.1	C36B—C35B—H35B	119.8
C37A—C36A—C35A	119.3 (5)	C37B—C36B—C35B	119.9 (5)
C37A—C36A—H36A	120.3	C37B—C36B—H36B	120.0
C35A—C36A—H36A	120.3	C35B—C36B—H36B	120.0
C36A—C37A—C32A	121.7 (5)	C36B—C37B—C32B	120.4 (5)
C36A—C37A—H37A	119.2	C36B—C37B—H37B	119.8
C32A—C37A—H37A	119.2	C32B—C37B—H37B	119.8
C39A—C38A—P1A	113.5 (3)	C39B—C38B—P1B	115.4 (3)
C39A—C38A—H38A	108.9	C39B—C38B—H38C	108.4
P1A—C38A—H38A	108.9	P1B—C38B—H38C	108.4
C39A—C38A—H38B	108.9	C39B—C38B—H38D	108.4
P1A—C38A—H38B	108.9	P1B—C38B—H38D	108.4
H38A—C38A—H38B	107.7	H38C—C38B—H38D	107.5
C44A—C39A—C40A	118.1 (5)	C40B—C39B—C44B	117.9 (5)
C44A—C39A—C38A	120.3 (5)	C40B—C39B—C38B	121.7 (4)
C40A—C39A—C38A	121.6 (5)	C44B—C39B—C38B	120.3 (5)
C41A—C40A—C39A	120.1 (5)	C39B—C40B—C41B	121.0 (5)
C41A—C40A—H40A	119.9	C39B—C40B—H40B	119.5
C39A—C40A—H40A	119.9	C41B—C40B—H40B	119.5
C42A—C41A—C40A	121.1 (5)	C42B—C41B—C40B	120.7 (6)
C42A—C41A—H41A	119.5	C42B—C41B—H41B	119.6
C40A—C41A—H41A	119.5	C40B—C41B—H41B	119.6
C43A—C42A—C41A	119.6 (5)	C41B—C42B—C43B	119.4 (5)
C43A—C42A—H42A	120.2	C41B—C42B—H42B	120.3
C41A—C42A—H42A	120.2	C43B—C42B—H42B	120.3
C42A—C43A—C44A	120.2 (5)	C42B—C43B—C44B	120.0 (5)
C42A—C43A—H43A	119.9	C42B—C43B—H43B	120.0
C44A—C43A—H43A	119.9	C44B—C43B—H43B	120.0
C43A—C44A—C39A	120.8 (5)	C43B—C44B—C39B	120.9 (5)
C43A—C44A—H44A	119.6	C43B—C44B—H44B	119.5
C39A—C44A—H44A	119.6	C39B—C44B—H44B	119.5
O1A—C45A—Ru1A	173.1 (5)	O1B—C45B—Ru1B	174.1 (4)
O2A—C46A—Ru1A	176.5 (5)	O2B—C46B—Ru1B	176.5 (5)
O3A—C47A—Ru1A	171.6 (4)	O3B—C47B—Ru1B	173.1 (5)
O4A—C48A—Ru2A	173.5 (4)	O4B—C48B—Ru2B	173.8 (5)
O5A—C49A—Ru2A	176.2 (5)	O5B—C49B—Ru2B	177.5 (5)
O6A—C50A—Ru2A	174.3 (4)	O6B—C50B—Ru2B	170.7 (4)
O7A—C51A—Ru3A	173.4 (4)	O7B—C51B—Ru3B	172.4 (5)
O8A—C52A—Ru3A	176.6 (5)	O8B—C52B—Ru3B	179.4 (5)
O9A—C53A—Ru3A	173.3 (4)	O9B—C53B—Ru3B	173.2 (4)
			
C47A—Ru1A—Ru2A—C49A	−55.4 (4)	C46B—Ru1B—Ru2B—C50B	55.2 (4)
C45A—Ru1A—Ru2A—C49A	117.4 (4)	C45B—Ru1B—Ru2B—C50B	−23.5 (2)
As1A—Ru1A—Ru2A—C49A	−149.5 (4)	C47B—Ru1B—Ru2B—C50B	161.0 (2)
Ru3A—Ru1A—Ru2A—C49A	22.5 (4)	As2B—Ru1B—Ru2B—C50B	−112.02 (15)
C47A—Ru1A—Ru2A—C48A	20.9 (2)	Ru3B—Ru1B—Ru2B—C50B	73.27 (15)
C45A—Ru1A—Ru2A—C48A	−166.3 (2)	C46B—Ru1B—Ru2B—C48B	−117.4 (4)
As1A—Ru1A—Ru2A—C48A	−73.19 (15)	C45B—Ru1B—Ru2B—C48B	163.9 (2)
Ru3A—Ru1A—Ru2A—C48A	98.80 (15)	C47B—Ru1B—Ru2B—C48B	−11.7 (2)
C47A—Ru1A—Ru2A—C50A	−161.0 (2)	As2B—Ru1B—Ru2B—C48B	75.34 (16)
C45A—Ru1A—Ru2A—C50A	11.8 (2)	Ru3B—Ru1B—Ru2B—C48B	−99.38 (16)
As1A—Ru1A—Ru2A—C50A	104.88 (15)	C46B—Ru1B—Ru2B—As1B	149.2 (4)
Ru3A—Ru1A—Ru2A—C50A	−83.13 (15)	C45B—Ru1B—Ru2B—As1B	70.53 (14)
C47A—Ru1A—Ru2A—As2A	110.44 (15)	C47B—Ru1B—Ru2B—As1B	−105.03 (15)
C45A—Ru1A—Ru2A—As2A	−76.75 (15)	As2B—Ru1B—Ru2B—As1B	−18.01 (2)
As1A—Ru1A—Ru2A—As2A	16.36 (2)	Ru3B—Ru1B—Ru2B—As1B	167.27 (2)
Ru3A—Ru1A—Ru2A—As2A	−171.65 (2)	C46B—Ru1B—Ru2B—Ru3B	−18.0 (4)
C47A—Ru1A—Ru2A—Ru3A	−77.91 (15)	C45B—Ru1B—Ru2B—Ru3B	−96.75 (14)
C45A—Ru1A—Ru2A—Ru3A	94.90 (15)	C47B—Ru1B—Ru2B—Ru3B	87.70 (15)
As1A—Ru1A—Ru2A—Ru3A	−171.99 (2)	As2B—Ru1B—Ru2B—Ru3B	174.72 (2)
C49A—Ru2A—Ru3A—C52A	15.2 (2)	C46B—Ru1B—Ru3B—C52B	−13.9 (2)
C48A—Ru2A—Ru3A—C52A	108.8 (2)	C45B—Ru1B—Ru3B—C52B	−107.8 (2)
C50A—Ru2A—Ru3A—C52A	−76.2 (2)	C47B—Ru1B—Ru3B—C52B	79.4 (2)
As2A—Ru2A—Ru3A—C52A	−152.18 (16)	As2B—Ru1B—Ru3B—C52B	158.95 (16)
Ru1A—Ru2A—Ru3A—C52A	−173.33 (15)	Ru2B—Ru1B—Ru3B—C52B	173.30 (15)
C49A—Ru2A—Ru3A—C51A	105.8 (2)	C46B—Ru1B—Ru3B—C53B	−107.0 (2)
C48A—Ru2A—Ru3A—C51A	−160.6 (2)	C45B—Ru1B—Ru3B—C53B	159.2 (2)
C50A—Ru2A—Ru3A—C51A	14.4 (2)	C47B—Ru1B—Ru3B—C53B	−13.7 (2)
As2A—Ru2A—Ru3A—C51A	−61.56 (16)	As2B—Ru1B—Ru3B—C53B	65.89 (16)
Ru1A—Ru2A—Ru3A—C51A	−82.71 (15)	Ru2B—Ru1B—Ru3B—C53B	80.24 (15)
C49A—Ru2A—Ru3A—C53A	−74.0 (2)	C46B—Ru1B—Ru3B—C51B	73.4 (2)
C48A—Ru2A—Ru3A—C53A	19.6 (2)	C45B—Ru1B—Ru3B—C51B	−20.5 (2)
C50A—Ru2A—Ru3A—C53A	−165.4 (2)	C47B—Ru1B—Ru3B—C51B	166.6 (2)
As2A—Ru2A—Ru3A—C53A	118.60 (16)	As2B—Ru1B—Ru3B—C51B	−113.79 (17)
Ru1A—Ru2A—Ru3A—C53A	97.45 (15)	Ru2B—Ru1B—Ru3B—C51B	−99.44 (16)
C49A—Ru2A—Ru3A—P1A	−144.6 (2)	C46B—Ru1B—Ru3B—P1B	146.8 (2)
C48A—Ru2A—Ru3A—P1A	−51.0 (2)	C45B—Ru1B—Ru3B—P1B	52.9 (2)
C50A—Ru2A—Ru3A—P1A	124.0 (2)	C47B—Ru1B—Ru3B—P1B	−120.0 (2)
As2A—Ru2A—Ru3A—P1A	47.96 (15)	As2B—Ru1B—Ru3B—P1B	−40.38 (17)
Ru1A—Ru2A—Ru3A—P1A	26.81 (13)	Ru2B—Ru1B—Ru3B—P1B	−26.04 (15)
C49A—Ru2A—Ru3A—Ru1A	−171.45 (16)	C46B—Ru1B—Ru3B—Ru2B	172.81 (16)
C48A—Ru2A—Ru3A—Ru1A	−77.84 (16)	C45B—Ru1B—Ru3B—Ru2B	78.95 (15)
C50A—Ru2A—Ru3A—Ru1A	97.14 (16)	C47B—Ru1B—Ru3B—Ru2B	−93.92 (16)
As2A—Ru2A—Ru3A—Ru1A	21.15 (5)	As2B—Ru1B—Ru3B—Ru2B	−14.35 (6)
C46A—Ru1A—Ru3A—C52A	−159.3 (4)	C49B—Ru2B—Ru3B—C52B	156.8 (4)
C47A—Ru1A—Ru3A—C52A	116.5 (4)	C50B—Ru2B—Ru3B—C52B	−120.2 (4)
C45A—Ru1A—Ru3A—C52A	−66.1 (4)	C48B—Ru2B—Ru3B—C52B	63.4 (4)
As1A—Ru1A—Ru3A—C52A	31.3 (4)	As1B—Ru2B—Ru3B—C52B	−39.9 (4)
Ru2A—Ru1A—Ru3A—C52A	15.9 (3)	Ru1B—Ru2B—Ru3B—C52B	−16.4 (4)
C46A—Ru1A—Ru3A—C51A	−79.3 (2)	C49B—Ru2B—Ru3B—C53B	78.1 (2)
C47A—Ru1A—Ru3A—C51A	−163.5 (2)	C50B—Ru2B—Ru3B—C53B	161.1 (2)
C45A—Ru1A—Ru3A—C51A	13.9 (2)	C48B—Ru2B—Ru3B—C53B	−15.3 (2)
As1A—Ru1A—Ru3A—C51A	111.30 (15)	As1B—Ru2B—Ru3B—C53B	−118.56 (15)
Ru2A—Ru1A—Ru3A—C51A	95.84 (14)	Ru1B—Ru2B—Ru3B—C53B	−95.07 (15)
C46A—Ru1A—Ru3A—C53A	103.9 (2)	C49B—Ru2B—Ru3B—C51B	−105.8 (2)
C47A—Ru1A—Ru3A—C53A	19.7 (2)	C50B—Ru2B—Ru3B—C51B	−22.8 (2)
C45A—Ru1A—Ru3A—C53A	−162.9 (2)	C48B—Ru2B—Ru3B—C51B	160.8 (2)
As1A—Ru1A—Ru3A—C53A	−65.45 (15)	As1B—Ru2B—Ru3B—C51B	57.47 (16)
Ru2A—Ru1A—Ru3A—C53A	−80.91 (15)	Ru1B—Ru2B—Ru3B—C51B	80.97 (15)
C46A—Ru1A—Ru3A—P1A	12.45 (18)	C49B—Ru2B—Ru3B—P1B	−13.29 (17)
C47A—Ru1A—Ru3A—P1A	−71.73 (15)	C50B—Ru2B—Ru3B—P1B	69.71 (15)
C45A—Ru1A—Ru3A—P1A	105.63 (16)	C48B—Ru2B—Ru3B—P1B	−106.70 (16)
As1A—Ru1A—Ru3A—P1A	−156.93 (5)	As1B—Ru2B—Ru3B—P1B	150.00 (5)
Ru2A—Ru1A—Ru3A—P1A	−172.38 (4)	Ru1B—Ru2B—Ru3B—P1B	173.50 (4)
C46A—Ru1A—Ru3A—Ru2A	−175.17 (17)	C49B—Ru2B—Ru3B—Ru1B	173.21 (17)
C47A—Ru1A—Ru3A—Ru2A	100.66 (15)	C50B—Ru2B—Ru3B—Ru1B	−103.79 (15)
C45A—Ru1A—Ru3A—Ru2A	−81.99 (16)	C48B—Ru2B—Ru3B—Ru1B	79.80 (16)
As1A—Ru1A—Ru3A—Ru2A	15.46 (4)	As1B—Ru2B—Ru3B—Ru1B	−23.50 (4)
C46A—Ru1A—As1A—C7A	−91.6 (2)	C49B—Ru2B—As1B—C7B	93.1 (2)
C47A—Ru1A—As1A—C7A	−3.4 (2)	C50B—Ru2B—As1B—C7B	4.4 (2)
C45A—Ru1A—As1A—C7A	175.3 (2)	C48B—Ru2B—As1B—C7B	−175.2 (2)
Ru2A—Ru1A—As1A—C7A	91.74 (18)	Ru1B—Ru2B—As1B—C7B	−91.41 (17)
Ru3A—Ru1A—As1A—C7A	78.52 (18)	Ru3B—Ru2B—As1B—C7B	−71.42 (17)
C46A—Ru1A—As1A—C1A	30.4 (2)	C49B—Ru2B—As1B—C1B	−27.7 (2)
C47A—Ru1A—As1A—C1A	118.6 (2)	C50B—Ru2B—As1B—C1B	−116.4 (2)
C45A—Ru1A—As1A—C1A	−62.7 (2)	C48B—Ru2B—As1B—C1B	64.1 (2)
Ru2A—Ru1A—As1A—C1A	−146.32 (16)	Ru1B—Ru2B—As1B—C1B	147.84 (17)
Ru3A—Ru1A—As1A—C1A	−159.54 (16)	Ru3B—Ru2B—As1B—C1B	167.82 (17)
C46A—Ru1A—As1A—C13A	144.3 (2)	C49B—Ru2B—As1B—C13B	−143.5 (2)
C47A—Ru1A—As1A—C13A	−127.5 (2)	C50B—Ru2B—As1B—C13B	127.8 (2)
C45A—Ru1A—As1A—C13A	51.2 (2)	C48B—Ru2B—As1B—C13B	−51.7 (2)
Ru2A—Ru1A—As1A—C13A	−32.36 (15)	Ru1B—Ru2B—As1B—C13B	32.06 (16)
Ru3A—Ru1A—As1A—C13A	−45.58 (16)	Ru3B—Ru2B—As1B—C13B	52.04 (17)
C49A—Ru2A—As2A—C20A	−67.3 (2)	C46B—Ru1B—As2B—C14B	−48.8 (2)
C48A—Ru2A—As2A—C20A	−160.7 (2)	C45B—Ru1B—As2B—C14B	45.0 (2)
C50A—Ru2A—As2A—C20A	24.2 (2)	C47B—Ru1B—As2B—C14B	−141.3 (2)
Ru3A—Ru2A—As2A—C20A	99.81 (18)	Ru3B—Ru1B—As2B—C14B	138.59 (17)
Ru1A—Ru2A—As2A—C20A	118.31 (17)	Ru2B—Ru1B—As2B—C14B	125.93 (17)
C49A—Ru2A—As2A—C14A	51.5 (2)	C46B—Ru1B—As2B—C20B	71.0 (2)
C48A—Ru2A—As2A—C14A	−41.9 (2)	C45B—Ru1B—As2B—C20B	164.7 (2)
C50A—Ru2A—As2A—C14A	143.0 (2)	C47B—Ru1B—As2B—C20B	−21.5 (2)
Ru3A—Ru2A—As2A—C14A	−141.37 (18)	Ru3B—Ru1B—As2B—C20B	−101.62 (19)
Ru1A—Ru2A—As2A—C14A	−122.88 (18)	Ru2B—Ru1B—As2B—C20B	−114.28 (18)
C49A—Ru2A—As2A—C13A	174.2 (2)	C46B—Ru1B—As2B—C13B	−170.6 (2)
C48A—Ru2A—As2A—C13A	80.8 (2)	C45B—Ru1B—As2B—C13B	−76.9 (2)
C50A—Ru2A—As2A—C13A	−94.3 (2)	C47B—Ru1B—As2B—C13B	96.9 (2)
Ru3A—Ru2A—As2A—C13A	−18.73 (17)	Ru3B—Ru1B—As2B—C13B	16.78 (18)
Ru1A—Ru2A—As2A—C13A	−0.23 (16)	Ru2B—Ru1B—As2B—C13B	4.12 (16)
C52A—Ru3A—P1A—C32A	−94.6 (2)	C52B—Ru3B—P1B—C32B	93.2 (2)
C51A—Ru3A—P1A—C32A	174.5 (2)	C53B—Ru3B—P1B—C32B	−173.3 (2)
C53A—Ru3A—P1A—C32A	−4.7 (2)	C51B—Ru3B—P1B—C32B	5.3 (2)
Ru2A—Ru3A—P1A—C32A	65.0 (2)	Ru1B—Ru3B—P1B—C32B	−67.5 (3)
Ru1A—Ru3A—P1A—C32A	88.89 (19)	Ru2B—Ru3B—P1B—C32B	−90.86 (19)
C52A—Ru3A—P1A—C26A	142.4 (2)	C52B—Ru3B—P1B—C26B	−144.6 (2)
C51A—Ru3A—P1A—C26A	51.5 (2)	C53B—Ru3B—P1B—C26B	−51.1 (2)
C53A—Ru3A—P1A—C26A	−127.7 (2)	C51B—Ru3B—P1B—C26B	127.5 (2)
Ru2A—Ru3A—P1A—C26A	−58.0 (2)	Ru1B—Ru3B—P1B—C26B	54.8 (3)
Ru1A—Ru3A—P1A—C26A	−34.08 (19)	Ru2B—Ru3B—P1B—C26B	31.4 (2)
C52A—Ru3A—P1A—C38A	22.4 (2)	C52B—Ru3B—P1B—C38B	−23.0 (2)
C51A—Ru3A—P1A—C38A	−68.5 (2)	C53B—Ru3B—P1B—C38B	70.5 (2)
C53A—Ru3A—P1A—C38A	112.3 (2)	C51B—Ru3B—P1B—C38B	−111.0 (2)
Ru2A—Ru3A—P1A—C38A	−177.95 (18)	Ru1B—Ru3B—P1B—C38B	176.30 (19)
Ru1A—Ru3A—P1A—C38A	−154.08 (17)	Ru2B—Ru3B—P1B—C38B	152.90 (17)
C7A—As1A—C1A—C2A	153.9 (4)	C7B—As1B—C1B—C6B	26.2 (5)
C13A—As1A—C1A—C2A	−99.4 (4)	C13B—As1B—C1B—C6B	−79.9 (4)
Ru1A—As1A—C1A—C2A	24.0 (4)	Ru2B—As1B—C1B—C6B	155.0 (4)
C7A—As1A—C1A—C6A	−30.5 (5)	C7B—As1B—C1B—C2B	−156.0 (4)
C13A—As1A—C1A—C6A	76.2 (4)	C13B—As1B—C1B—C2B	97.9 (4)
Ru1A—As1A—C1A—C6A	−160.4 (4)	Ru2B—As1B—C1B—C2B	−27.2 (4)
C6A—C1A—C2A—C3A	0.9 (8)	C6B—C1B—C2B—C3B	0.4 (8)
As1A—C1A—C2A—C3A	176.7 (4)	As1B—C1B—C2B—C3B	−177.5 (4)
C1A—C2A—C3A—C4A	−0.8 (8)	C1B—C2B—C3B—C4B	0.0 (8)
C2A—C3A—C4A—C5A	0.2 (8)	C2B—C3B—C4B—C5B	−0.4 (8)
C3A—C4A—C5A—C6A	0.5 (8)	C3B—C4B—C5B—C6B	0.5 (8)
C4A—C5A—C6A—C1A	−0.4 (8)	C4B—C5B—C6B—C1B	−0.2 (8)
C2A—C1A—C6A—C5A	−0.2 (8)	C2B—C1B—C6B—C5B	−0.3 (8)
As1A—C1A—C6A—C5A	−175.8 (4)	As1B—C1B—C6B—C5B	177.5 (4)
C1A—As1A—C7A—C8A	−65.2 (4)	C1B—As1B—C7B—C8B	62.4 (4)
C13A—As1A—C7A—C8A	−166.3 (4)	C13B—As1B—C7B—C8B	164.7 (4)
Ru1A—As1A—C7A—C8A	64.5 (4)	Ru2B—As1B—C7B—C8B	−65.8 (4)
C1A—As1A—C7A—C12A	117.6 (4)	C1B—As1B—C7B—C12B	−118.9 (4)
C13A—As1A—C7A—C12A	16.5 (5)	C13B—As1B—C7B—C12B	−16.6 (5)
Ru1A—As1A—C7A—C12A	−112.7 (4)	Ru2B—As1B—C7B—C12B	113.0 (4)
C12A—C7A—C8A—C9A	−1.4 (8)	C12B—C7B—C8B—C9B	1.2 (8)
As1A—C7A—C8A—C9A	−178.6 (4)	As1B—C7B—C8B—C9B	−180.0 (4)
C7A—C8A—C9A—C10A	1.3 (8)	C7B—C8B—C9B—C10B	−0.3 (8)
C8A—C9A—C10A—C11A	0.0 (9)	C8B—C9B—C10B—C11B	−0.9 (8)
C9A—C10A—C11A—C12A	−1.3 (9)	C9B—C10B—C11B—C12B	1.2 (9)
C10A—C11A—C12A—C7A	1.2 (8)	C10B—C11B—C12B—C7B	−0.3 (8)
C8A—C7A—C12A—C11A	0.1 (8)	C8B—C7B—C12B—C11B	−0.9 (8)
As1A—C7A—C12A—C11A	177.2 (4)	As1B—C7B—C12B—C11B	−179.7 (4)
C20A—As2A—C13A—As1A	−148.4 (2)	C7B—As1B—C13B—As2B	94.8 (3)
C14A—As2A—C13A—As1A	109.8 (3)	C1B—As1B—C13B—As2B	−160.8 (3)
Ru2A—As2A—C13A—As1A	−21.7 (3)	Ru2B—As1B—C13B—As2B	−35.6 (3)
C7A—As1A—C13A—As2A	−92.1 (3)	C14B—As2B—C13B—As1B	−112.5 (3)
C1A—As1A—C13A—As2A	163.7 (3)	C20B—As2B—C13B—As1B	143.7 (2)
Ru1A—As1A—C13A—As2A	38.6 (3)	Ru1B—As2B—C13B—As1B	16.9 (3)
C20A—As2A—C14A—C19A	−56.5 (5)	C20B—As2B—C14B—C19B	−122.3 (4)
C13A—As2A—C14A—C19A	48.2 (5)	C13B—As2B—C14B—C19B	132.3 (4)
Ru2A—As2A—C14A—C19A	175.7 (4)	Ru1B—As2B—C14B—C19B	6.0 (5)
C20A—As2A—C14A—C15A	118.7 (4)	C20B—As2B—C14B—C15B	53.7 (5)
C13A—As2A—C14A—C15A	−136.7 (4)	C13B—As2B—C14B—C15B	−51.7 (5)
Ru2A—As2A—C14A—C15A	−9.2 (5)	Ru1B—As2B—C14B—C15B	−178.0 (4)
C19A—C14A—C15A—C16A	−2.7 (8)	C19B—C14B—C15B—C16B	−3.2 (9)
As2A—C14A—C15A—C16A	−177.9 (4)	As2B—C14B—C15B—C16B	−179.3 (5)
C14A—C15A—C16A—C17A	1.6 (8)	C14B—C15B—C16B—C17B	2.0 (10)
C15A—C16A—C17A—C18A	−0.4 (8)	C15B—C16B—C17B—C18B	−0.1 (10)
C16A—C17A—C18A—C19A	0.2 (9)	C16B—C17B—C18B—C19B	−0.6 (9)
C17A—C18A—C19A—C14A	−1.3 (9)	C15B—C14B—C19B—C18B	2.5 (8)
C15A—C14A—C19A—C18A	2.5 (9)	As2B—C14B—C19B—C18B	178.5 (4)
As2A—C14A—C19A—C18A	177.7 (5)	C17B—C18B—C19B—C14B	−0.6 (8)
C14A—As2A—C20A—C21A	97.1 (5)	C14B—As2B—C20B—C21B	−127.7 (4)
C13A—As2A—C20A—C21A	−8.4 (5)	C13B—As2B—C20B—C21B	−20.8 (5)
Ru2A—As2A—C20A—C21A	−132.9 (4)	Ru1B—As2B—C20B—C21B	103.4 (4)
C14A—As2A—C20A—C25A	−79.1 (4)	C14B—As2B—C20B—C25B	54.5 (4)
C13A—As2A—C20A—C25A	175.4 (4)	C13B—As2B—C20B—C25B	161.4 (4)
Ru2A—As2A—C20A—C25A	50.9 (4)	Ru1B—As2B—C20B—C25B	−74.4 (4)
C25A—C20A—C21A—C22A	−2.1 (8)	C25B—C20B—C21B—C22B	0.2 (8)
As2A—C20A—C21A—C22A	−178.2 (4)	As2B—C20B—C21B—C22B	−177.6 (4)
C20A—C21A—C22A—C23A	1.2 (9)	C20B—C21B—C22B—C23B	−0.1 (9)
C21A—C22A—C23A—C24A	0.9 (9)	C21B—C22B—C23B—C24B	−0.1 (9)
C22A—C23A—C24A—C25A	−2.2 (9)	C22B—C23B—C24B—C25B	0.1 (9)
C23A—C24A—C25A—C20A	1.3 (8)	C23B—C24B—C25B—C20B	0.0 (9)
C21A—C20A—C25A—C24A	0.9 (8)	C21B—C20B—C25B—C24B	−0.1 (8)
As2A—C20A—C25A—C24A	177.3 (4)	As2B—C20B—C25B—C24B	177.8 (5)
C32A—P1A—C26A—C31A	−150.9 (4)	C32B—P1B—C26B—C31B	149.0 (4)
C38A—P1A—C26A—C31A	103.7 (4)	C38B—P1B—C26B—C31B	−106.2 (4)
Ru3A—P1A—C26A—C31A	−23.4 (5)	Ru3B—P1B—C26B—C31B	21.8 (5)
C32A—P1A—C26A—C27A	37.1 (5)	C32B—P1B—C26B—C27B	−34.8 (5)
C38A—P1A—C26A—C27A	−68.4 (4)	C38B—P1B—C26B—C27B	70.0 (5)
Ru3A—P1A—C26A—C27A	164.6 (3)	Ru3B—P1B—C26B—C27B	−161.9 (3)
C31A—C26A—C27A—C28A	−0.4 (8)	C31B—C26B—C27B—C28B	0.1 (8)
P1A—C26A—C27A—C28A	171.9 (4)	P1B—C26B—C27B—C28B	−176.2 (4)
C26A—C27A—C28A—C29A	−0.7 (8)	C26B—C27B—C28B—C29B	−0.4 (8)
C27A—C28A—C29A—C30A	1.1 (8)	C27B—C28B—C29B—C30B	0.8 (8)
C28A—C29A—C30A—C31A	−0.4 (8)	C28B—C29B—C30B—C31B	−1.0 (8)
C29A—C30A—C31A—C26A	−0.7 (8)	C27B—C26B—C31B—C30B	−0.3 (8)
C27A—C26A—C31A—C30A	1.1 (8)	P1B—C26B—C31B—C30B	176.1 (4)
P1A—C26A—C31A—C30A	−171.2 (4)	C29B—C30B—C31B—C26B	0.7 (8)
C26A—P1A—C32A—C37A	47.6 (4)	C26B—P1B—C32B—C33B	−47.7 (4)
C38A—P1A—C32A—C37A	151.2 (4)	C38B—P1B—C32B—C33B	−151.0 (4)
Ru3A—P1A—C32A—C37A	−84.6 (4)	Ru3B—P1B—C32B—C33B	85.6 (4)
C26A—P1A—C32A—C33A	−139.7 (4)	C26B—P1B—C32B—C37B	136.7 (4)
C38A—P1A—C32A—C33A	−36.0 (5)	C38B—P1B—C32B—C37B	33.4 (5)
Ru3A—P1A—C32A—C33A	88.1 (4)	Ru3B—P1B—C32B—C37B	−90.0 (4)
C37A—C32A—C33A—C34A	−0.7 (8)	C37B—C32B—C33B—C34B	−0.7 (7)
P1A—C32A—C33A—C34A	−173.5 (4)	P1B—C32B—C33B—C34B	−176.5 (4)
C32A—C33A—C34A—C35A	0.3 (9)	C32B—C33B—C34B—C35B	0.2 (8)
C33A—C34A—C35A—C36A	0.0 (9)	C33B—C34B—C35B—C36B	0.2 (8)
C34A—C35A—C36A—C37A	0.0 (8)	C34B—C35B—C36B—C37B	−0.1 (8)
C35A—C36A—C37A—C32A	−0.4 (8)	C35B—C36B—C37B—C32B	−0.4 (8)
C33A—C32A—C37A—C36A	0.7 (8)	C33B—C32B—C37B—C36B	0.8 (8)
P1A—C32A—C37A—C36A	173.7 (4)	P1B—C32B—C37B—C36B	176.5 (4)
C32A—P1A—C38A—C39A	−173.0 (4)	C32B—P1B—C38B—C39B	167.8 (4)
C26A—P1A—C38A—C39A	−66.9 (4)	C26B—P1B—C38B—C39B	63.2 (4)
Ru3A—P1A—C38A—C39A	64.1 (4)	Ru3B—P1B—C38B—C39B	−69.9 (4)
P1A—C38A—C39A—C44A	−104.1 (5)	P1B—C38B—C39B—C40B	100.8 (5)
P1A—C38A—C39A—C40A	73.6 (5)	P1B—C38B—C39B—C44B	−82.6 (5)
C44A—C39A—C40A—C41A	4.3 (7)	C44B—C39B—C40B—C41B	−1.5 (7)
C38A—C39A—C40A—C41A	−173.5 (5)	C38B—C39B—C40B—C41B	175.1 (5)
C39A—C40A—C41A—C42A	−3.0 (8)	C39B—C40B—C41B—C42B	0.5 (8)
C40A—C41A—C42A—C43A	−1.0 (8)	C40B—C41B—C42B—C43B	0.1 (9)
C41A—C42A—C43A—C44A	3.7 (8)	C41B—C42B—C43B—C44B	0.3 (9)
C42A—C43A—C44A—C39A	−2.4 (8)	C42B—C43B—C44B—C39B	−1.4 (8)
C40A—C39A—C44A—C43A	−1.6 (7)	C40B—C39B—C44B—C43B	2.0 (8)
C38A—C39A—C44A—C43A	176.2 (5)	C38B—C39B—C44B—C43B	−174.7 (5)

### Hydrogen-bond geometry (Å, °)

**Table:**

Cg1, Cg2, Cg3 and Cg4 are the centroids of the C1B–C6B, C1A–C6A, C26A–C31A and C26B–C31B benzene rings, respectively.

**Table:**

*D*—H···*A*	*D*—H	H···*A*	*D*···*A*	*D*—H···*A*
C16A—H16A···O6B	0.93	2.59	3.189 (6)	123
C30A—H30A···O7A^i^	0.93	2.58	3.261 (6)	130
C42B—H42B···O9B^ii^	0.93	2.58	3.241 (7)	129
C28A—H28A···Cg1^iii^	0.93	2.72	3.570 (7)	152
C28B—H28B···Cg2^iv^	0.93	2.87	3.648 (7)	142
C40A—H40A···Cg3	0.93	2.77	3.474 (7)	133
C44B—H44B···Cg4	0.93	2.94	3.615 (7)	130

Symmetry codes: (i) −*x*+2, −*y*+2, −*z*+1; (ii) −*x*, −*y*+1, −*z*+1; (iii) *x*+1, −*y*+1/2, *z*−1/2; (iv) *x*−1, −*y*+1/2, *z*−3/2.
